# Texture Analyzer-Derived SeDeM-ODT Extension for Bisoprolol Fumarate Orodispersible Tablets: Formulation Discrimination Within a Pharmacopoeial Disintegration-Compliant Space

**DOI:** 10.3390/pharmaceutics18080940

**Published:** 2026-07-30

**Authors:** Çağla Afşin, Sevinç Şahbaz, Setenay Özer-Önder, Timuçin Uğurlu

**Affiliations:** 1 Institute of Health Sciences, Marmara University, Mehmet Genç Külliyesi, Dragos 34865, İstanbul, Türkiye; caglaafsin@marun.edu.tr; 2 Department of Pharmaceutical Technology, Faculty of Pharmacy, Marmara University, Başıbüyük 34854, İstanbul, Türkiye; sevren@marmara.edu.tr (S.Ş.); setenay.ozer@marmara.edu.tr (S.Ö.-Ö.)

**Keywords:** SeDeM-ODT expert system, texture analyzer, orodispersible tablets, low-volume liquid disintegration, swelling efficiency, residue height, structural transition efficiency, central composite design, bisoprolol fumarate

## Abstract

**Background/Objectives:** Conventional SeDeM-ODT screening relies on physicochemical properties and endpoint disintegration tests, which may have limited discriminatory power among formulations that already meet pharmacopoeial disintegration requirements. This study aimed to extend SeDeM-ODT by incorporating texture analyzer-derived descriptors of low-volume liquid disintegration behavior. **Methods:** Bisoprolol fumarate was used as a low-dose model drug. Selected excipients were characterized using SeDeM and conventional SeDeM-ODT approaches. Texture analyzer distance–time profiles were used to derive swelling efficiency (SE), residue height (RH), and structural transition efficiency (STE), which were converted into SeDeM-compatible parameters. Orodispersible tablets were developed using a two-factor central composite design and evaluated for mechanical properties, pharmacopoeial disintegration, comparative dissolution performance, texture analyzer behavior, and supportive Heckel parameters. **Results:** All formulations met the pharmacopoeial disintegration criteria and showed rapid drug release under the applied dissolution conditions. Conventional endpoint-based responses showed limited discriminatory value within the investigated formulation space. In contrast, SE, RH, and STE differentiated formulation-dependent swelling, residual structural persistence, and transition toward structural collapse under low-volume liquid controlled-force conditions. MCC-rich formulations generally retained greater residual structure, whereas lactose-rich and/or higher-superdisintegrant formulations showed lower residual persistence. Comparative kinetic fitting and Heckel analysis supported these interpretations but did not independently establish a definitive disintegration mechanism. **Conclusions:** Incorporating low-volume liquid texture analyzer-derived parameters into SeDeM-ODT improved the comparative interpretation of excipient and formulation behavior. These findings suggest that pharmacopoeial disintegration compliance may coexist with distinct structural pathways not fully captured by conventional endpoints.

## 1. Introduction

Orodispersible tablets (ODTs) are solid oral dosage forms designed to disintegrate rapidly in the oral cavity before swallowing, thereby improving ease of administration and patient acceptability, particularly in pediatric, geriatric, dysphagic, and non-compliant patient populations [1,2,3,4]. According to the European Pharmacopeia, orodispersible tablets should disintegrate within 3 min; however, faster, more predictable disintegration is generally desirable to ensure patient comfort and consistent product performance [5]. In addition to rapid disintegration, ODTs must exhibit sufficient mechanical strength to withstand manufacturing, packaging, handling, and administration. Therefore, ODT development requires a careful balance between rapid liquid-mediated breakdown and adequate tablet robustness [3,6].

Direct compression is one of the most attractive manufacturing approaches for ODTs because of its simplicity, cost-effectiveness, limited processing steps, and suitability for scale-up [7,8]. However, the success of direct compression strongly depends on the functional properties of the active pharmaceutical ingredient and excipients [9]. Powder flowability, compactability, compressibility, lubricity, dilution potential, and disintegration behavior all contribute to the manufacturability and performance of the final dosage form [10]. This is particularly important in low-drug-loaded ODT formulations, where the excipient system predominantly governs tablet structure, porosity, mechanical integrity, and disintegration behavior. Consequently, rational excipient selection is essential for developing robust directly compressed ODTs.

The SeDeM Expert System provides a systematic preformulation tool for evaluating the suitability of powders for direct compression [11]. By transforming experimentally determined powder properties into normalized radius values, the SeDeM approach enables visual and numerical assessment of critical powder attributes, including dimensional characteristics, compressibility, flowability, lubricity/stability, and lubricity/dosage [12]. The SeDeM-ODT Expert System further extends this framework by incorporating a disgregability factor, making it more relevant for materials intended for orally disintegrating dosage forms [10,13]. This makes SeDeM and SeDeM-ODT useful tools for identifying functional limitations in APIs and excipients prior to formulation development.

Nevertheless, the conventional disgregability factor in SeDeM-ODT is mainly based on effervescence/dispersion time and pharmacopoeial disintegration testing performed with and without a disk [14,15]. These tests are valuable for confirming whether a material or formulation meets predefined disintegration requirements, but they may provide limited mechanistic information. In particular, endpoint-based or observation-dependent measurements may fail to distinguish materials or formulations that show similar disintegration times but differ in swelling capacity, structural persistence, liquid-mediated weakening, or collapse behavior. This limitation becomes especially relevant when comparing superdisintegrants or when evaluating formulations that already comply with pharmacopoeial disintegration requirements.

Texture analyzer-based disintegration testing offers an opportunity to obtain a more quantitative and mechanistically informative description of tablet disintegration [16]. Under controlled-force and low-volume liquid conditions, the texture analyzer records distance–time profiles that reflect dynamic structural changes during liquid uptake, swelling, structural transition, and residual plateau formation. Previous studies have demonstrated the usefulness of texture analysis for measuring disintegration-related parameters such as swelling distance, onset and end of disintegration, disintegration rate, and residue height [16,17]. However, the integration of texture analyzer-derived descriptors into a SeDeM-compatible framework remains limited. Such integration may improve the discriminatory capacity of SeDeM-ODT by converting profile-derived disintegration behavior into normalized parameters that can be compared with conventional SeDeM indices. In addition, comparative kinetic evaluation of distance–time profiles may support the interpretation of how swelling and structural transition progress over time, without assigning a definitive disintegration or drug-release mechanism.

A quality-by-design-oriented formulation strategy also requires systematic evaluation of how formulation variables affect critical quality attributes [18]. Central composite design (CCD) is widely used to investigate formulation factors, interactions, and nonlinear effects while reducing experimental burden [19,20]. In ODT development, CCD can be used to evaluate how diluent composition and superdisintegrant concentration influence tablet mechanical strength, pharmacopoeial disintegration, dissolution performance, and texture analyzer-derived disintegration behavior. However, when formulations exhibit similar pharmacopoeial disintegration and dissolution outcomes, conventional tests may not be sufficiently discriminatory within the compliant design space. In this context, texture analyzer-derived parameters may provide additional insight into formulation-dependent structural behavior.

Bisoprolol fumarate was selected as a low-dose model drug because its limited drug loading allowed formulation performance to be governed predominantly by the excipient matrix. This study aimed to develop a texture analyzer-derived extension of the SeDeM-ODT framework capable of providing profile-based discrimination among directly compressed ODT formulations that already satisfy the pharmacopoeial disintegration requirement. The novelty of the proposed approach lies in transforming swelling efficiency (SE), residue height (RH), and structural transition efficiency (STE) into SeDeM-compatible parameters, thereby complementing conventional endpoint-based disgregability assessment with quantitative information on swelling development, residual structural persistence, and structural transition under low-volume liquid conditions.

## 2. Materials and Methods

### 2.1. Materials

Bisoprolol fumarate was provided by World Medicine (Türkiye). Polyplasdone^®^ XL and Polyplasdone^®^ XL-10 (crospovidone; Ashland, USA), Vivapur^®^ 200 (microcrystalline cellulose; JRS Pharma GmbH & Co. KG, Germany), Avicel^®^ PH 102 (microcrystalline cellulose; IFF Pharma Solutions, USA), and Tablettose^®^ 80 (α-lactose monohydrate; MEGGLE GmbH & Co. KG, Germany) were kindly supplied by Ashland Türkiye. Vivasol^®^ (croscarmellose sodium; JRS Pharma GmbH & Co. KG, Germany) was kindly supplied by Bilim İlaç (Türkiye). Magnesium stearate was obtained from Sigma-Aldrich (St. Louis, MO, USA). All materials were of pharmaceutical grade and were used as received without further modification. Distilled water was used throughout the experiments involving aqueous media.

### 2.2. SeDeM and SeDeM-ODT Evaluation

The suitability of the investigated excipients and the active pharmaceutical ingredient for direct compression was evaluated using the SeDeM Expert System. Bisoprolol fumarate, microcrystalline cellulose grades (Avicel^®^ PH 102 and Vivapur^®^ 200), and α-lactose monohydrate were characterized according to the original SeDeM methodology.

Since the primary functionality of superdisintegrants is directly associated with tablet disintegration behavior, Polyplasdone^®^ XL, Polyplasdone^®^ XL-10, and Vivasol^®^ were additionally evaluated using the SeDeM-ODT Expert System, which incorporates the disgregability factor into the conventional SeDeM framework [14].

Powder characterization was performed using the parameters summarized in Table 1. Experimentally determined values were transformed into normalized radius values (r) ranging from 0 to 10 using the corresponding SeDeM and SeDeM-ODT conversion equations [21]. The obtained radius values were used to construct radial diagrams and calculate the Parameter Index (IP), Parameter Profile Index (IPP), and the corresponding indices, namely the Index of Good Compressibility (IGC) for the original SeDeM profile and the Index of Good Compressibility and Bucodispersibility (IGCB) for the SeDeM-ODT profile [22].

The definitions, equations, and acceptance criteria of the calculated indices are presented in Table 2. Radius values of 5 or greater were considered acceptable according to the SeDeM and SeDeM-ODT methodologies. The overall suitability of each material was subsequently assessed based on the calculated IP, IPP, and IGCB values.

The conventional SeDeM-ODT results were used as the reference endpoint-based disgregability evaluation, whereas the texture analyzer-derived extension of the disgregability factor is described separately in Section 2.4.

### 2.3. Texture Analyzer-Based Characterization of Disintegration Behavior

Although the SeDeM-ODT Expert System includes a disgregability factor for the evaluation of materials intended for orally disintegrating tablets, the conventional tests included in this factor, namely effervescence/dispersion time, disintegration time with disk, and disintegration time without disk, provided limited discriminatory information for differentiating the investigated superdisintegrants. Therefore, a texture analyzer-based approach was used to obtain a more quantitative and mechanistically informative characterization of disintegration behavior under low-volume, liquid-controlled, force conditions [17,29].

Texture analyzer measurements were initially performed on Polyplasdone^®^ XL, Polyplasdone^®^ XL-10, and Vivasol^®^ to compare the disintegration behavior of the investigated superdisintegrants. In addition, Vivapur^®^ 200 and α-lactose monohydrate were included as non-superdisintegrant reference materials representing distinct disintegration and structural persistence tendencies. Microcrystalline cellulose was selected as a swelling-prone and structure-forming reference material, whereas α-lactose monohydrate was selected as a soluble and fragmentation-prone reference material [30,31]. These reference materials were included to provide mechanistic contrast for the interpretation of texture analyzer-derived distance–time profiles.

Pure-material compacts used for texture analyzer testing were prepared by compressing 500 mg of each material at 150 bar (15 MPa) using a laboratory-scale hydraulic press equipped with 12 mm flat-faced punches. A dwell time of 10 s was applied. This compression pressure was used as a standardized comparative condition for the preparation of the investigated pure-material compacts. Following ejection, the compacts were visually examined for capping, lamination, cracking, edge fracture, and other visible structural defects. The compacts were stored in closed containers under ambient laboratory conditions and evaluated approximately 24 h after preparation.

**Figure 1:**
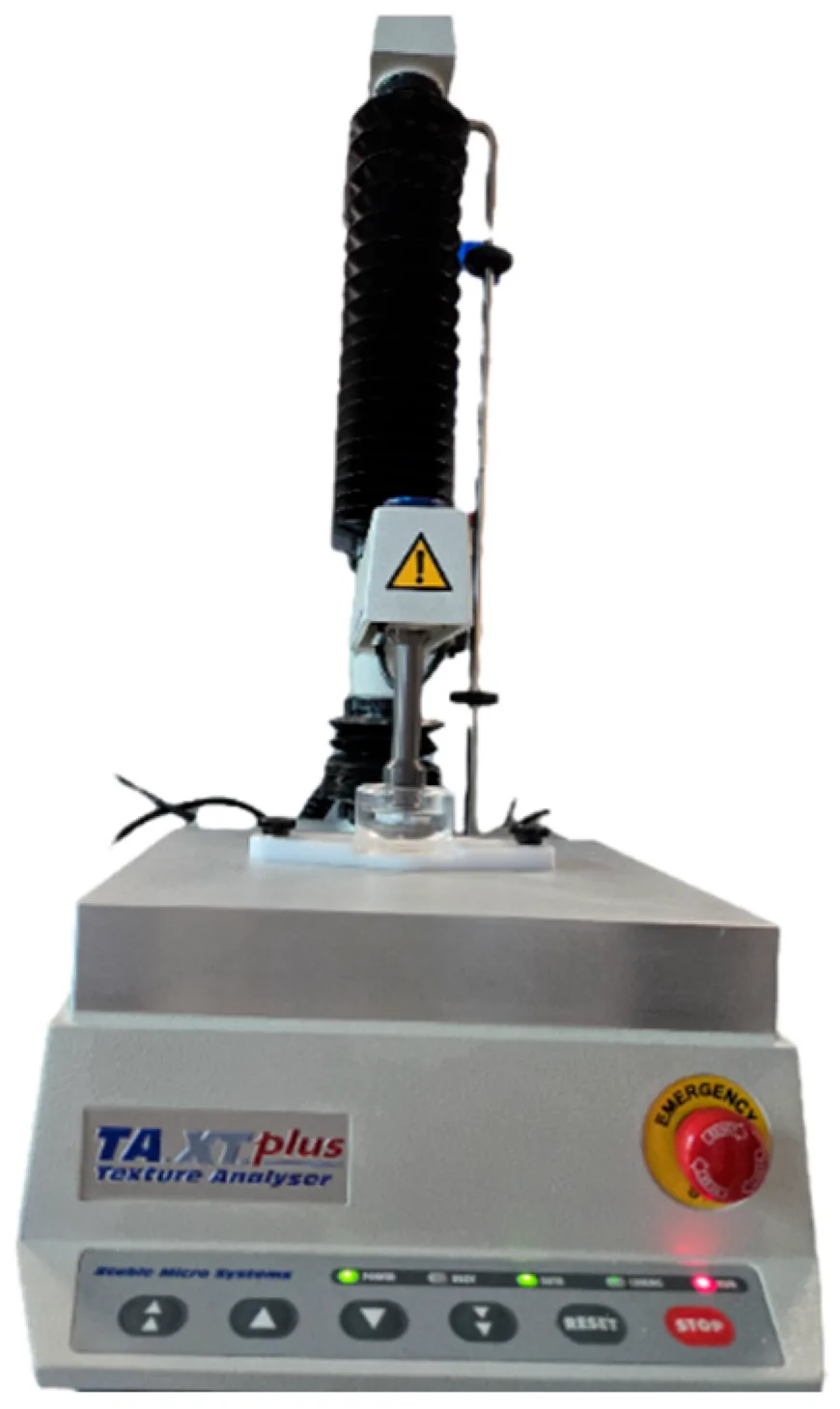
Texture analyzer-based low-volume liquid disintegration test setup used for the characterization of disintegration-related distance–time profiles. The tablet sample was attached to the lower surface of the probe and lowered into a vessel containing 6 mL of distilled water maintained at 37 ± 0.5 °C. Measurements were performed under controlled-force conditions using a TA.XT Plus Texture Analyzer.

The measurements were carried out using a texture analyzer (TA.XT Plus, Stable Micro Systems, Godalming, UK) equipped with a tablet disintegration rig. The apparatus was calibrated using a 5 kg load cell. Each sample was attached to the lower surface of the probe head using double-sided adhesive tape. The probe head carrying the sample was then lowered into a Perspex vessel containing 6 mL of distilled water maintained at 37 ± 0.5 °C. The test was performed in compression mode using the following parameters: pre-test speed, 2 mm/s; test speed, 3 mm/s; post-test speed, 10 mm/s; target mode, force; applied force, 60 g; and trigger force, 5 g. Measurements were performed in triplicate for each sample. The experimental setup used for texture analyzer-based low-volume liquid disintegration test is shown in Figure 1.

The distance–time profiles obtained from the texture analyzer were exported and further processed to derive custom disintegration descriptors. The profiles were interpreted according to characteristic regions corresponding to swelling, structural transition, and residual persistence. The initial distance at the beginning of the test was defined as D_0_. Point A was defined as the maximum swelling point, corresponding to the highest distance value reached during the swelling phase. The distance and time values at Point A were recorded as maximum swelling distance (D_max_) and time to maximum swelling (tA), respectively.

Point B was defined operationally as the first point after tA at which the distance signal reached a stable residual region, determined by a relative distance change of ≤1% over a 30 s interval. The distance and time values at Point B were recorded as residue height (RH) and time to residual plateau (tB), respectively. Positive RH values were interpreted as indicating remaining upward structural persistence relative to the initial reference level, whereas negative RH values indicated collapse or downward displacement after the main disintegration event.

Based on these primary profile descriptors, swelling efficiency (SE), residue height (RH), and structural transition efficiency (STE) were calculated. SE was calculated as the ratio of D_max_ to tA and was used to describe the rate-normalized swelling response of the sample after contact with the aqueous medium.

SE = Dmax/tA(1)

where Dmax is the maximum swelling distance and tA is the time required to reach maximum swelling. This ratio distinguishes a large swelling response reached rapidly from the same response developing over a longer period, which is particularly relevant to rapidly disintegrating systems. When no measurable upward swelling maximum was observed, Dmax, tA, and SE were assigned a value of 0.

RH was used directly as an indicator of residual structural persistence or collapse at the first stable residual region after the main structural transition. Lower or more negative RH values were interpreted as indicating lower residual structure or greater collapse, whereas higher positive RH values indicated more persistent structure after liquid exposure.

STE was calculated by normalizing the residue height by the time required to reach the stable residual region:

STE = −RH/tB(2)

where RH is the residue height at Point B and tB is the time corresponding to the residual plateau. The inclusion of tB allows comparable residual structural levels reached over different time periods to be distinguished. The negative sign was used so that lower or more negative RH values, which indicate greater collapse or lower residual structural persistence, corresponded to higher STE values. Therefore, higher STE values were interpreted as reflecting a more efficient transition toward structural collapse or loss of residual structure under the applied low-volume liquid conditions, whereas lower or negative STE values indicated greater residual structural persistence.

**Figure 2:**
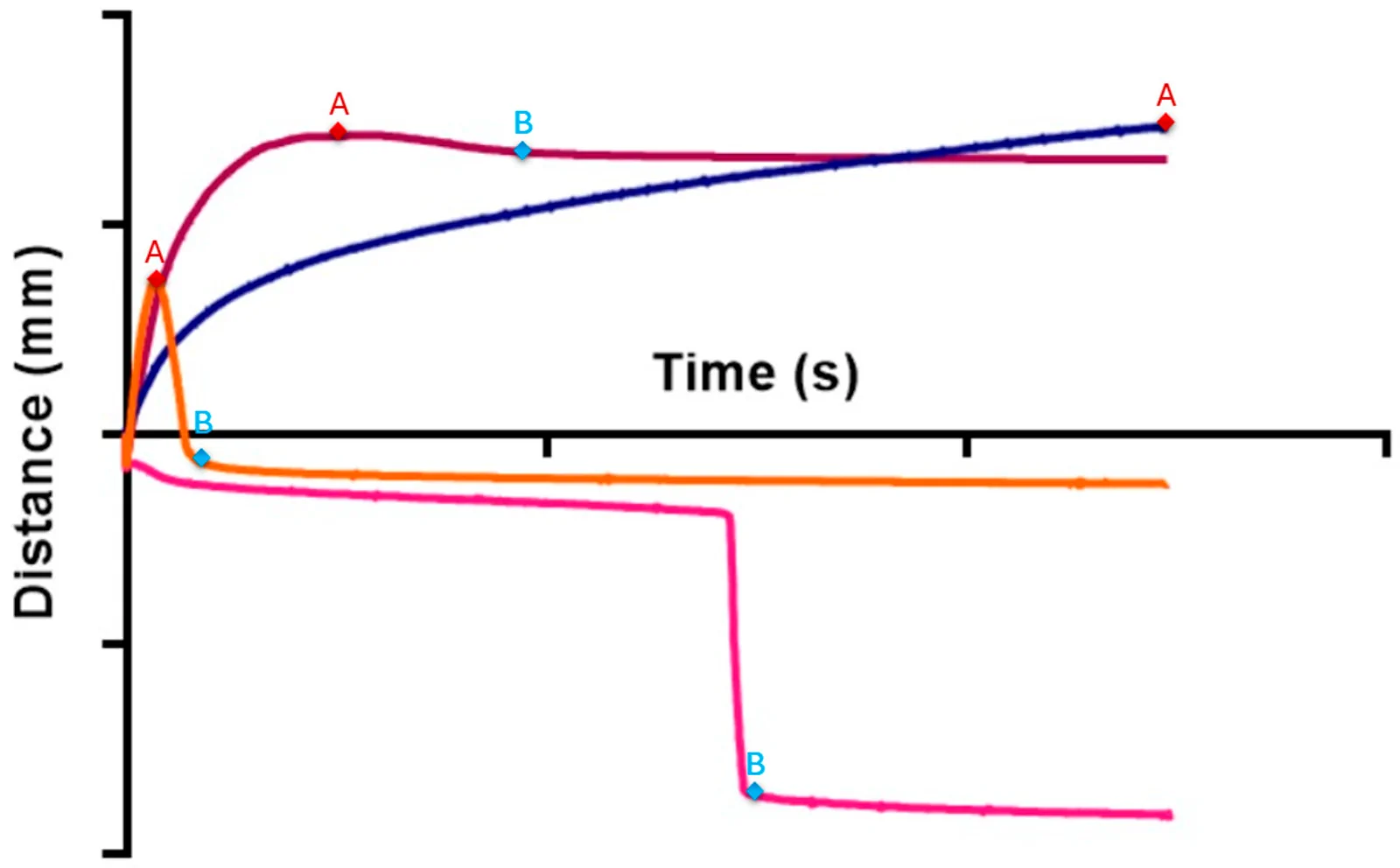
Representative texture analyzer distance–time profiles used for the extraction of custom disintegration descriptors. Point A represents the maximum swelling point, from which maximum swelling distance (D_max_) and time to maximum swelling (tA) were obtained. Point B represents the first stable point after tA, defined as the point at which the relative change between the distance value at that time point and the value recorded 30 s later was ≤1%. The time and distance values at Point B were recorded as time to residual plateau (tB) and residue height (RH), respectively. Swelling efficiency (SE) was calculated as D_max_/tA, whereas structural transition efficiency (STE) was calculated as −RH/tB. The differently colored lines illustrate possible distance–time profile patterns and are included to demonstrate the identification of Points A and B across profiles with different swelling and structural transition behavior; they do not represent specific materials or formulations.

The extracted descriptors were subsequently used to develop SeDeM-compatible disgregability parameters. The definition of Points A and B and the extraction of the primary profile descriptors are illustrated in Figure 2.

### 2.4. Development of Texture Analyzer-Derived SeDeM-Compatible Disgregability Parameters

The texture analyzer-derived descriptors were transformed into SeDeM-compatible radius values to provide a quantitative extension of the disgregability factor. This transformation was performed to allow swelling, residual structure, and structural transition behavior obtained from distance–time profiles to be expressed on the same normalized 0–10 scale used in the SeDeM and SeDeM-ODT systems.

For this purpose, the experimentally observed descriptor values obtained from the investigated superdisintegrants and the selected non-superdisintegrant reference materials were used to define practical normalization limits. Polyplasdone^®^ XL, Polyplasdone^®^ XL-10, and Vivasol^®^ were used to represent the functional range of superdisintegrant behavior, whereas Vivapur^®^ 200 and α-lactose monohydrate were included as reference materials with contrasting swelling, dissolution, and residual structure-forming tendencies. These limits were therefore used as empirical normalization boundaries rather than pharmacopoeial acceptance criteria.

Three texture analyzer-derived descriptors were selected for SeDeM-compatible transformation: swelling efficiency (SE), structural transition efficiency (STE), and residue height (RH). SE was used to describe the rate-normalized swelling response after contact with the aqueous medium. STE was used to describe the time-normalized transition toward loss of residual structure, as calculated from the residue height at Point B and the corresponding time to residual plateau. RH was used to quantify the remaining structural level after the main disintegration event.

The original texture analyzer-derived descriptor values were denoted as SE, STE, and RH, whereas their normalized SeDeM-compatible radius values were denoted as rSE, rSTE, and rRH, respectively. The r-prefixed terms therefore refer only to the transformed 0–10 radius values used in the TA-extended SeDeM-ODT profile and radial diagrams.

For SE and STE, higher values were considered to indicate more favorable disintegration-related behavior and were therefore transformed using direct scaling. In contrast, RH was transformed using inverse scaling because higher positive RH values indicate greater residual structural persistence, whereas lower or more negative RH values indicate lower residual structure or greater collapse. The proposed transformation equations and normalization limits are summarized in Table 3.

Calculated radius values below 0 or above 10 were truncated to 0 and 10, respectively, in accordance with the normalized SeDeM radius scale. The resulting radius values, namely rSE, rSTE, and rRH, were incorporated into the disgregability factor as texture analyzer-derived alternatives to the conventional endpoint-based disgregability parameters.

### 2.5. Comparative Kinetic Modeling of Texture Analyzer Profiles

In addition to the SeDeM-compatible transformation of the texture analyzer-derived descriptors, the original distance–time profiles were further evaluated using comparative kinetic modeling. This analysis was performed using the exported texture analyzer data and was intended to describe the time-dependent evolution of the profiles during swelling and structural transition. The kinetic models were not used to assign a definitive drug-release or disintegration mechanism, but rather to compare whether the profile evolution showed mathematical similarity to constant-rate, first-order-like, diffusion/penetration-like, geometry-related, or empirical power-law patterns.

For each texture analyzer profile, the initial distance at the beginning of the test was defined as D_0_. Point A was defined as the maximum swelling point, and the corresponding distance and time values were recorded as D_max_ and tA, respectively. After Point A, Point B was determined according to the stability criterion described in Section 2.3. The time and distance values at this point were recorded as tB and residue height (RH), respectively.

The kinetic analysis was performed separately for the swelling phase and the structural transition phase. The swelling phase was defined as the region from D_0_ to Point A, and the structural transition phase as the region from Point A to Point B. To minimize the influence of initial signal noise, abrupt fluctuations, and plateau instability, kinetic fitting was performed using the most representative 60% region of each selected profile segment.

The selected distance–time data were fitted in Microsoft Excel using linearized forms of commonly used empirical kinetic models, namely the zero-order, first-order, Higuchi, Hixson–Crowell, and Korsmeyer–Peppas models [32,33,34,35,36]. Although these models are traditionally used for drug release and dissolution data, they were applied here only to compare the shapes of texture analyzer distance–time profile segments, not to infer a definitive drug-release or disintegration mechanism. For each model, the apparent fitting constant and R^2^ value were calculated, and the model with the highest R^2^ was considered to provide the best empirical fit for the corresponding segment. The equations and interpretations are summarized in Table 4.

The resulting model fits were interpreted only as comparative mathematical representations of texture analyzer profile evolution. Therefore, the terms zero-order, first-order, Higuchi, Hixson–Crowell, and Korsmeyer–Peppas were used in this study to indicate mathematical similarity to these empirical kinetic patterns, rather than to establish a definitive disintegration or drug-release mechanism.

### 2.6. Experimental Design and Formulation Development

A two-factor central composite design (CCD) was employed to investigate the effects of formulation variables on the critical quality attributes and texture analyzer-derived disintegration descriptors of bisoprolol fumarate orodispersible tablets [19,37,38]. The experimental design was generated using Design-Expert^®^ software, version 13 (Stat-Ease Inc., Minneapolis, MN, USA).

The independent variables were the microcrystalline cellulose/lactose monohydrate ratio in the diluent phase and the Polyplasdone^®^ XL concentration. The diluent phase consisted of Vivapur^®^ 200 and Tablettose^®^ 80. The MCC/LM factor represented the percentage of Vivapur^®^ 200 within the total diluent fraction, while the remaining portion of the diluent phase was completed with Tablettose^®^ 80. Polyplasdone^®^ XL was selected as the superdisintegrant and was expressed as a percentage of the total tablet weight.

The CCD consisted of 13 formulations, including factorial points, axial points, and center-point replicates. The investigated levels of the independent variables are presented in Table 5. Magnesium stearate was kept constant at 1% (*w/w*) in all formulations.

Each tablet contained 5 mg bisoprolol fumarate and had a total target weight of 300 mg. Magnesium stearate was fixed at 3 mg per tablet. The amount of Polyplasdone^®^ XL was calculated according to the CCD level, and the remaining tablet mass was distributed between Vivapur^®^ 200 and Tablettose^®^ 80 according to the selected MCC/LM level.

The response variables included tensile strength, pharmacopoeial disintegration time, dissolution performance, and the texture analyzer-derived descriptors swelling efficiency (SE), residue height (RH), and structural transition efficiency (STE). The texture analyzer-derived responses were included to determine whether the proposed descriptors could discriminate between formulations exhibiting similar conventional pharmacopoeial performance.

### 2.7. Preparation of Orodispersible Tablets

Orodispersible tablets were prepared by direct compression using a hydraulic press (Kaan Kalıp, İstanbul, Türkiye) equipped with 12 mm flat-faced punches, each weighing 300 mg. For each formulation, bisoprolol fumarate, Vivapur^®^ 200, Tablettose^®^ 80, and Polyplasdone^®^ XL were accurately weighed according to the experimental design and mixed for 5 min using a laboratory-scale V-mixer. Magnesium stearate was then added as the lubricant, and the blend was mixed for an additional 5 min. Tablets were compressed at a constant compression pressure of 200 bar (20 MPa) using a hydraulic press. The prepared tablets were stored in closed containers under ambient laboratory conditions until further characterization. The quantitative compositions of the formulations are presented in Table 6.

### 2.8. Evaluation of Orodispersible Tablets

The prepared orodispersible tablets were evaluated in terms of physical properties, mechanical performance, pharmacopoeial disintegration, dissolution performance, and texture analyzer-derived attributes. Results were expressed as mean ± standard deviation.

Tablet weight variation was determined by individually weighing 20 tablets of each formulation using an analytical balance (Shimadzu ATX-224, Kyoto, Japan). Tablet thickness and diameter were measured for 10 tablets using a digital caliper (Mitutoyo CD-15CPX, Japan). Tablet crushing strength was determined for 10 tablets using a tablet hardness tester (Holland C50, Nottingham, UK).

Tensile strength was calculated from tablet crushing force, diameter, and thickness using Equation (3) [39]:

(3) $$\sigma_{t}=\frac{2F}{\pi Dh}$$

where σ_t_ is the tensile strength, F is the crushing force, D is the tablet diameter, and h is the tablet thickness.

Friability was determined using a friability tester (Aymes, İstanbul, Türkiye). A pre-weighed sample of 10 tablets was rotated at 25 rpm for 4 min, corresponding to 100 revolutions. After the test, the tablets were dedusted and reweighed. Friability was calculated as the percentage weight loss relative to the initial tablet weight.

Pharmacopoeial disintegration time was determined in accordance with Ph. Eur. 2.9.1 using a disintegration tester (Pharmatest PTZ Auto, Hainburg, Germany) [24]. Distilled water was used as the disintegration medium and maintained at 37 ± 0.5 °C. The test was performed using three tablets from each formulation, and disintegration time was recorded as the time required for complete disintegration of the tablets. The results were evaluated in accordance with the European Pharmacopeia requirements for orodispersible tablets [24].

The UV spectrophotometric method was validated for bisoprolol fumarate quantification in the dissolution medium. The calibration curve was linear over the concentration range of 5–25 µg/mL, with a regression equation of y = 0.0361x + 0.0023 (R^2^ = 0.999998), where y is the absorbance at 225 nm and x is the concentration in µg/mL. The intra-day precision, expressed as relative standard deviation (RSD), ranged from 0.996% to 1.737% across nine replicate measurements at each concentration level, well below the commonly accepted limit of 2.0%. Additionally, no significant interference was observed from the excipient matrix in placebo solutions. These validation results confirmed that the method was reliable and suitable for the comparative dissolution evaluation of the bisoprolol fumarate orodispersible tablets.

In vitro dissolution studies were performed in triplicate using USP Apparatus II (paddle method) with a dissolution tester (AT7 Smart, Sotax, Switzerland). The dissolution medium consisted of 500 mL purified water maintained at 37 ± 0.5 °C, and the paddle speed was set at 50 rpm. Each tablet containing 5 mg bisoprolol fumarate was placed into the dissolution vessel.

Samples of 5 mL were withdrawn at 5, 10, 15, and 30 min and replaced with an equal volume of fresh dissolution medium maintained at the same temperature. The collected samples were analyzed by UV-visible spectrophotometry at 225 nm using a UV-visible spectrophotometer (UV 5, Mettler Toledo, Italy). Purified water was used as the instrumental blank, and placebo tablet solutions prepared under the same dissolution conditions were used as matrix blanks to minimize possible interference from excipients.

The amount of bisoprolol fumarate released was calculated using a previously constructed calibration curve. Cumulative percentage drug release was calculated based on the labeled amount of 5 mg bisoprolol fumarate per tablet and corrected for sample withdrawal and replacement. Q5 drug release percentage was additionally explored as an early-release response in the central composite design because it reflects the initial release performance of the orodispersible formulations.

Although the official monograph specifies a dissolution medium volume of 900 mL and an acceptance criterion of not less than 75% release at 45 min, the medium volume was reduced to 500 mL in the present study to obtain a quantifiable UV absorbance response for the low-dose formulation [40]. Therefore, the dissolution data were interpreted as comparative rapid-release performance with reference to the pharmacopoeial Q criterion, rather than as a formal monograph-equivalent dissolution compliance test. The UV spectrophotometric method used for bisoprolol fumarate quantification in dissolution samples was validated under the applied dissolution conditions in terms of linearity, accuracy, precision, and specificity against placebo matrix interference. The sampling time points were selected to characterize the early release profile of the orodispersible tablets.

Texture analyzer evaluation of the CCD formulations was performed using the method described in Section 2.3. The resulting distance–time profiles were processed to calculate swelling efficiency (SE), residue height (RH), and structural transition efficiency (STE). SE was calculated as Dmax/tA, where Dmax is the maximum swelling distance and tA is the time required to reach maximum swelling. STE was calculated as −RH/tB, where RH is the residue height at Point B, and tB is the time corresponding to the residual plateau. These parameters were used to determine whether the proposed texture analyzer-derived descriptors could distinguish formulation-level disintegration behavior within a pharmacopoeial disintegration-compliant formulation space.

### 2.9. Compression Behavior and Heckel Analysis

The compression and densification behavior of the individual excipients was evaluated using thickness-based apparent density measurements and Heckel analysis. The purpose of this analysis was to support the interpretation of texture analyzer-derived disintegration behavior by comparing the relative densification tendencies of the investigated materials under the same compression conditions.

Pure excipient compacts were prepared from Vivapur^®^ 200, Avicel^®^ PH 102, Tablettose^®^ 80, Polyplasdone^®^ XL, Polyplasdone^®^ XL-10, and Vivasol^®^. For each material, 500 mg of powder was compressed using 12 mm flat-faced punches and a hydraulic press (Kaan Kalıp, İstanbul, Türkiye). Compression was performed at incremental pressures of 5, 10, 15, 20, 25, 30, 35, and 40 MPa, corresponding to 50, 100, 150, 200, 250, 300, 350, and 400 bar, respectively. A dwell time of 10 s was applied at each compression pressure, and all measurements were performed in triplicate.

After ejection, the compact thickness was measured to determine the apparent density. Because all compacts were made using the same punch diameter and powder mass, relative densification was assessed by comparing their volumes. Since this method relies on out-of-die thickness measurements and an experimentally determined reference thickness instead of true density, the Heckel parameters derived are understood as relative figures rather than absolute material constants [41].

The Heckel relationship was used to describe the densification behavior of the powders during compression and is expressed as [42]:

(4) $$ln\left[\frac{1}{1-D}\right]=KP+A$$

Here, D represents the relative density of the compact at a specific pressure P, with K and A being constants obtained from the slope and intercept of the linear part of the Heckel plot. In this study, D was calculated from the thickness-based apparent relative density using the compact prepared at 40 MPa, the highest pressure investigated, as an internal normalization reference. This reference was not assumed to represent true density, complete densification, or a zero-porosity state, and further densification above 40 MPa cannot be excluded.

Porosity $\left(\varepsilon \right)$ is defined by the void fraction within the powder bed and was calculated from apparent density $\left(\rho_{A}\right)$ and reference density $\left(\rho_{T}\right)$ as follows:

(5) $${\varepsilon =1-\rho }_{A}{/\rho }_{T}=1-D$$

The apparent density $\left(\rho_{A}\right)$ was calculated by dividing the compact mass by its apparent volume $\left(V_{A}\right)$, which was determined from the measured compact height and punch area after ejection using the out-of-die method. The relative density $\left(D\right)$ was calculated as:

(6) $$D=\frac{\rho_{A}}{\rho_{T}}=\frac{\left(\frac{m}{V_{A}}\right)}{\left(\frac{m}{V_{T}}\right)}=V_{T}/V_{A}$$

For cylindrical compacts prepared using the same punch diameter, the ratio of volumes simplifies to the ratio of thicknesses:

(7) $$\frac{V_{T}}{V_{A}}=\frac{h_{T}}{h_{A}}$$

where $h_{T}$ is the minimum compact thickness obtained at 40 MPa and $h_{A}$ is the compact thickness measured at a given compression pressure.

Heckel plots were constructed by plotting ln[1/(1−D)] against applied compression pressure. The slope $\left(K\right)$ of the linear portion was used to calculate the apparent yield pressure $\left(P_{y}\right)$:

(8) $$P_{y}=\frac{1}{K}$$

Lower $P_{y}$ values were interpreted as indicating a greater tendency toward plastic densification under the applied compression conditions, whereas higher $P_{y}$ values indicated greater resistance to densification and/or a more fragmentation-dominant compression behavior [43].

Heckel analysis was performed to provide mechanistic support for the texture analyzer findings by linking excipient deformation behavior with the observed differences in structural persistence and residue formation.

### 2.10. Statistical Analysis and Model Evaluation

Statistical analysis of the central composite design was performed using Design-Expert^®^ software, version 13 (Stat-Ease Inc., Minneapolis, MN, USA). The effects of the independent variables, namely MCC/LM ratio and Polyplasdone^®^ XL concentration, on the selected response variables were evaluated by analysis of variance (ANOVA).

The experimental responses included tensile strength, swelling efficiency (SE), residue height (RH), structural transition efficiency (STE), and pharmacopoeial disintegration time. In vitro dissolution performance was evaluated over 30 min using the cumulative drug release values obtained at 5, 10, 15, and 30 min. The Q5 drug release percentage was additionally explored as an early-release response in the CCD model because it represents the initial dissolution behavior of the orodispersible formulations.

Linear, two-factor interaction (2FI), and quadratic models were evaluated for each response. The most appropriate model was selected based on model significance, lack-of-fit analysis, adjusted coefficient of determination (adjusted R^2^), predicted coefficient of determination (predicted R^2^), and adequate precision. Model terms were considered statistically significant when *p* < 0.05 [44].

The lack of fit was assessed to ensure that the selected model accurately represented the experimental data rather than pure error. Adequate precision measured the signal-to-noise ratio, with values above 4 indicating a desirable level for exploring the design space. If the overall model was not statistically significant or if the predicted R^2^ values were low, the corresponding response was considered to have limited predictive ability within the studied design space [45].

The polynomial models fitted to the data were used to create response surface and contour plots for responses deemed statistically significant. When relevant, regression equations were provided in both coded and actual factors to help interpret factor effects and predict responses within the experimental space.

## 3. Results

### 3.1. SeDeM Evaluation of Bisoprolol Fumarate and Selected Excipients

The SeDeM Expert System was used to evaluate the direct-compression suitability of bisoprolol fumarate and selected excipients prior to formulation development [46].

The calculated radius values, factor scores, and indexes are presented in Table 7.

Among the investigated superdisintegrants, Polyplasdone^®^ XL showed the highest IPP and IGC values, with an IPP of 5.19 and an IGC of 4.94. Polyplasdone^®^ XL-10 showed an IPP of 4.19 and an IGC of 3.99, while Vivasol showed the lowest values among the superdisintegrants, with an IPP of 3.17 and an IGC of 3.02.

For the diluent group, Tablettose^®^ 80 showed the highest original SeDeM index, with an IPP of 5.95 and an IGC of 5.67. Vivapur^®^ 200 and Avicel PH 102 showed comparable SeDeM profiles, with IPP values of 4.90 and 4.83 and IGC values of 4.67 and 4.60, respectively. Bisoprolol fumarate showed an IPP of 5.63 and an IGC of 5.36.

**Figure 3:**
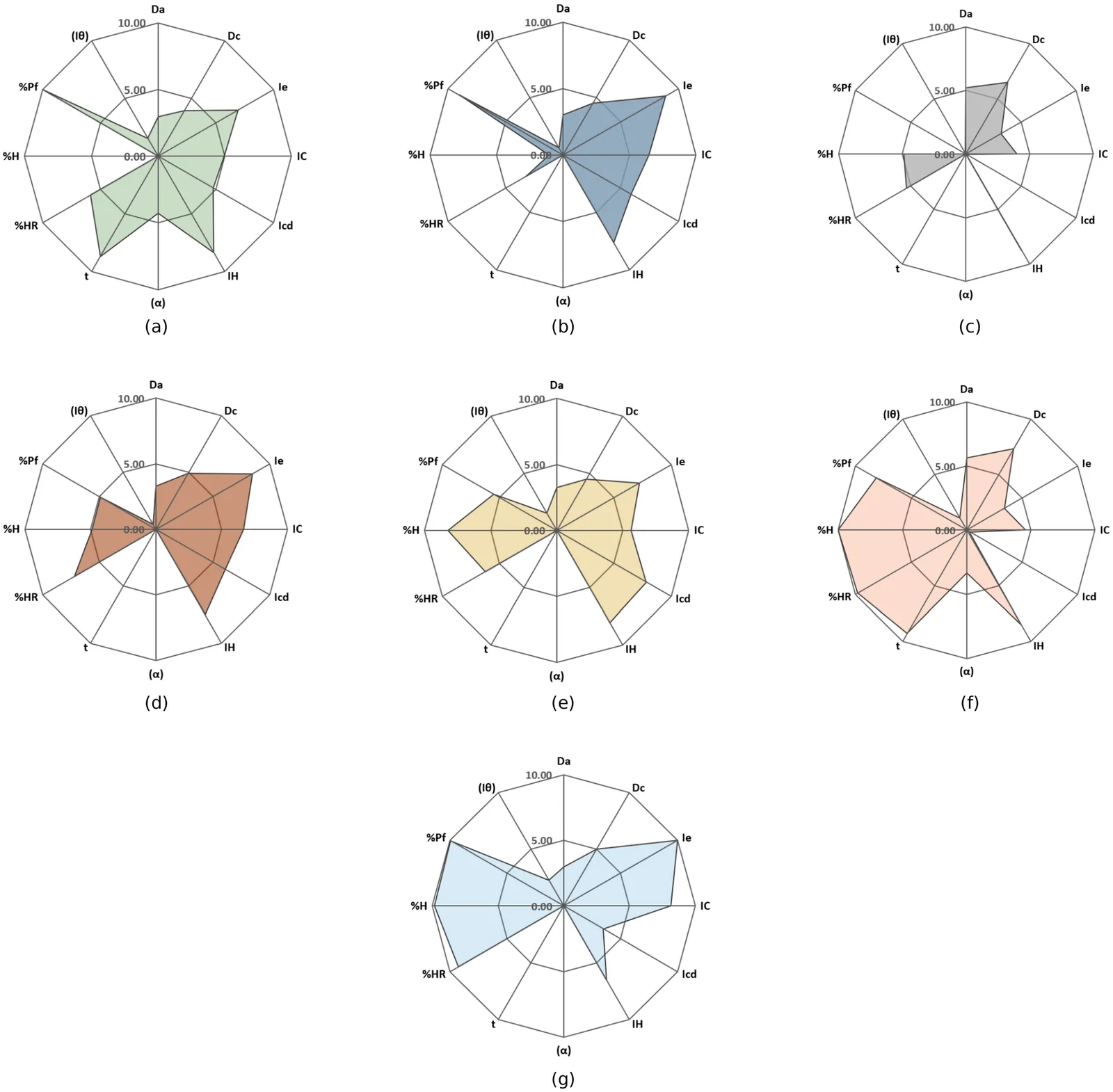
Comparative SeDeM diagrams of the evaluated materials: superdisintegrants [(**a**) Polyplasdone^®^ XL, (**b**) Polyplasdone^®^ XL-10, and (**c**) Vivasol], diluents [(**d**) Vivapur^®^ 200, (**e**) Avicel^®^ PH 102, and (**f**) Tablettose^®^ 80], and the active pharmaceutical ingredient [(**g**) bisoprolol fumarate].

The corresponding SeDeM diagrams are shown in Figure 3.

### 3.2. Conventional SeDeM-ODT Evaluation of the Investigated Superdisintegrants

The investigated superdisintegrants were further evaluated using the conventional SeDeM-ODT approach, which includes the disgregability factor in addition to the original SeDeM parameters [8].

The calculated radius values, disgregability factor scores, Parameter Index (IP), Parameter Profile Index (IPP), and Index of Good Compressibility and Bucodispersibility (IGCB) values are presented in Table 8.

The inclusion of the conventional disgregability parameters decreased the original SeDeM-ODT indices of the investigated superdisintegrants. Polyplasdone^®^ XL showed the highest conventional SeDeM-ODT profile among the three materials, with an IP of 0.40, an IPP of 4.48, and an IGCB of 4.35. Polyplasdone^®^ XL-10 showed an IP of 0.33, an IPP of 3.35, and an IGCB of 3.25. Vivasol showed the lowest conventional SeDeM-ODT profile, with an IP of 0.27, an IPP of 2.80, and an IGCB of 2.72.

For the disgregability factor, Polyplasdone^®^ XL showed the highest value among the investigated superdisintegrants, with a factor score of 1.67. Vivasol showed a disgregability factor score of 1.35, whereas Polyplasdone^®^ XL-10 showed a value of 0.00 under the conventional SeDeM-ODT evaluation conditions.

**Figure 4:**
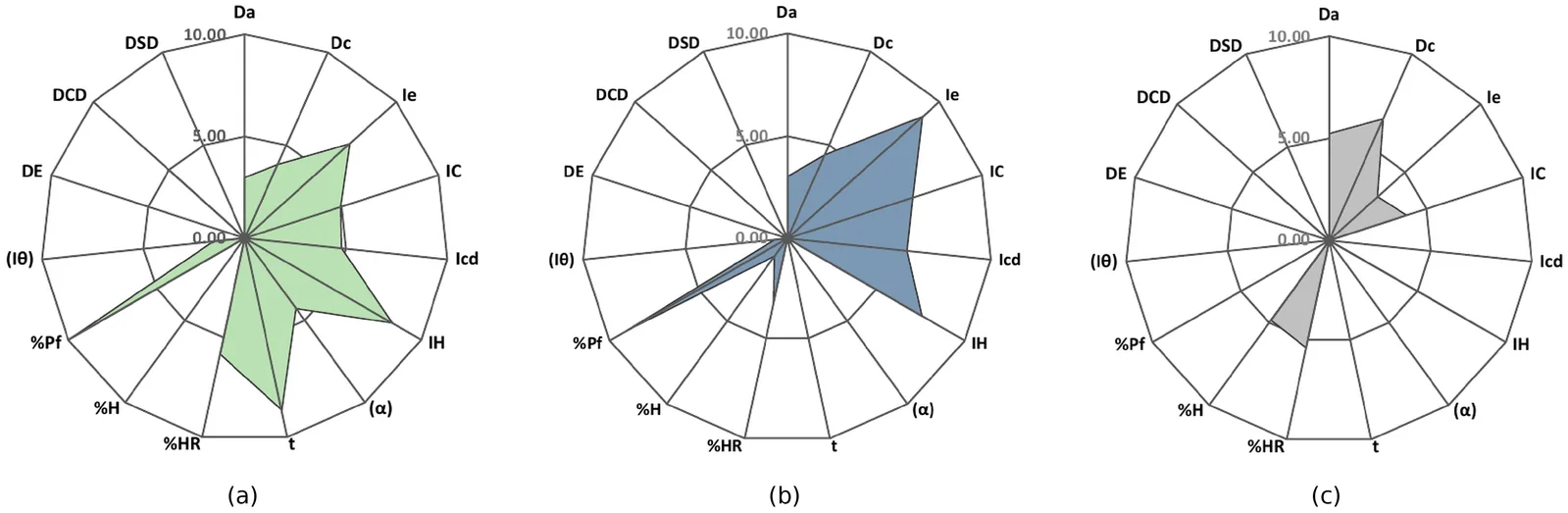
Conventional SeDeM-ODT diagrams of the evaluated superdisintegrants: (**a**) Polyplasdone^®^ XL, (**b**) Polyplasdone^®^ XL-10, and (**c**) Vivasol.

The corresponding conventional SeDeM-ODT diagrams are shown in Figure 4.

### 3.3. Texture Analyzer Profiles of Pure Superdisintegrants and Reference Excipients

All pure-material compacts were successfully ejected and handled without visible capping, lamination, cracking, edge fracture, or other loss of structural integrity.

Texture analyzer measurements were performed on the investigated superdisintegrants and selected reference excipients to obtain distance–time profiles under low-volume liquid controlled-force conditions.

**Figure 5:**
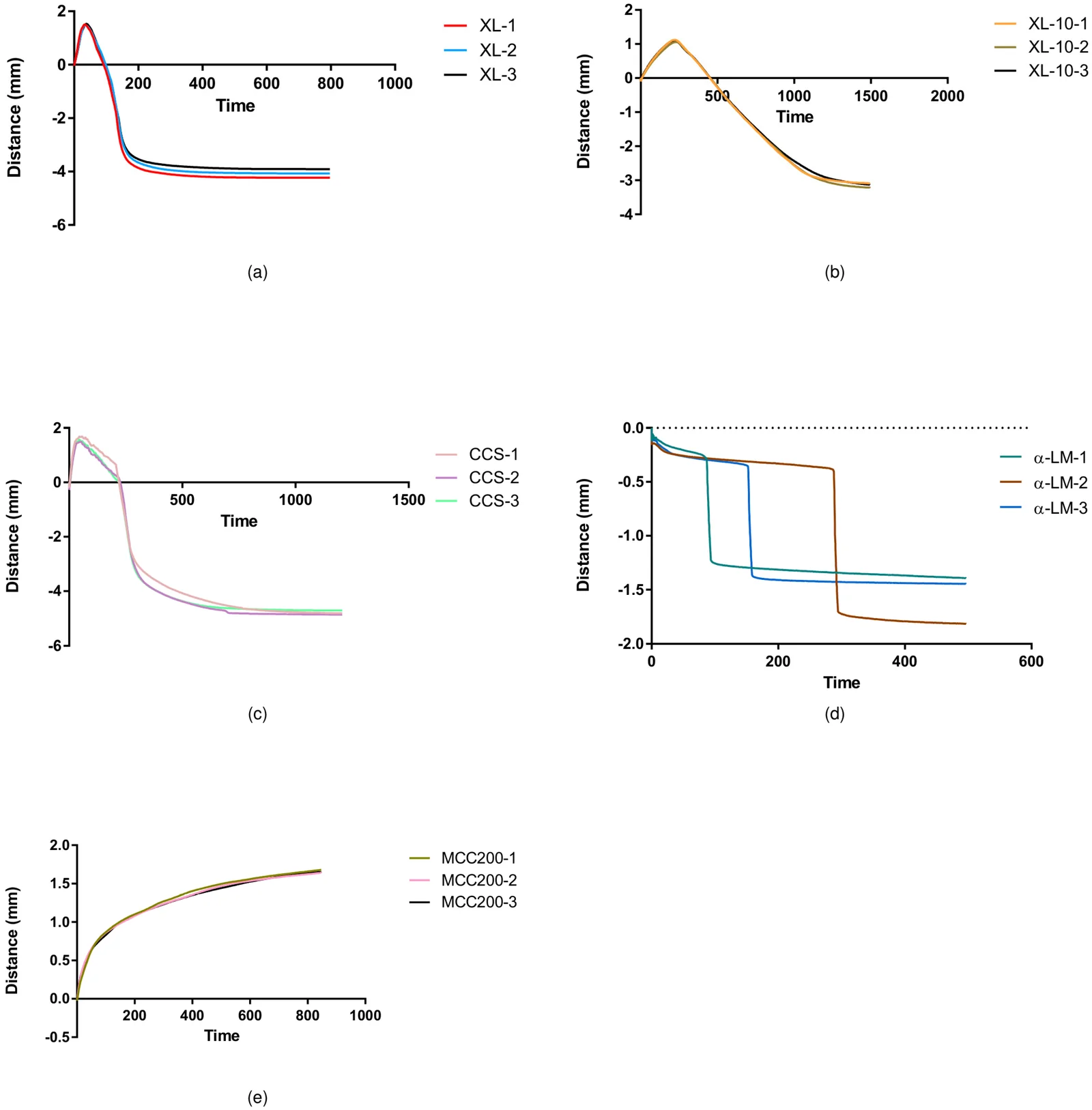
Texture analyzer distance–time profiles of pure superdisintegrants and reference excipients: (**a**) Polyplasdone^®^ XL/crospovidone type A, (**b**) Polyplasdone^®^ XL-10/crospovidone type B, (**c**) Vivasol/croscarmellose sodium, (**d**) Tablettose^®^ 80/α-lactose monohydrate, and (**e**) Vivapur^®^ 200/MCC 200. Each profile represents triplicate measurements obtained under low-volume liquid controlled-force conditions.

The extracted texture analyzer-derived descriptors are presented in Table 9, and the representative profiles are shown in Figure 5.

SE and STE were calculated from individual texture analyzer profiles before statistical summarization. Distinct distance–time profiles were obtained for the investigated materials. Among the superdisintegrants, Polyplasdone^®^ XL showed a D_max_ of 1.5020 ± 0.0300 mm and reached maximum swelling at 34.4670 ± 2.5720 s, resulting in the highest swelling efficiency value among the evaluated materials (SE = 0.0436). Vivasol^®^ showed a similar maximum swelling distance, with a D_max_ of 1.5960 ± 0.0950 mm, but reached the maximum swelling point later, with a tA of 47.3330 ± 3.5350 s. Polyplasdone^®^ XL-10 showed a lower D_max_ value of 1.0930 ± 0.0320 mm and a markedly longer tA of 218.9330 ± 5.8590 s, resulting in a lower SE value of 0.0050.

Residue height values also differed among the superdisintegrants. Vivasol^®^ showed the lowest RH value, at −4.4610 ± 0.1860 mm, followed by Polyplasdone^®^ XL at −4.0250 ± 0.0890 mm and Polyplasdone^®^ XL-10 at −2.5580 ± 0.8210 mm. The calculated STE values were 0.0142 for Polyplasdone^®^ XL, 0.0024 for Polyplasdone^®^ XL-10, and 0.0072 for Vivasol^®^.

The reference excipients showed contrasting profile behavior. Tablettose^®^ 80 did not show a measurable upward swelling maximum under the applied conditions; therefore, D_max_, tA, and SE were recorded as 0.0000. However, it showed a negative RH value of −1.4480 ± 0.2660 mm and an STE value of 0.0069. In contrast, Vivapur^®^ 200 showed a Dmax of 1.5410 ± 0.0170 mm, a prolonged tA of 599.0000 ± 0.7210 s, and a positive RH value of 1.5690 ± 0.0380 mm. Its calculated STE value was −0.0025.

These extracted descriptors were subsequently transformed into SeDeM-compatible radius values for the TA-extended SeDeM-ODT evaluation.

### 3.4. Texture Analyzer-Derived SeDeM-ODT Evaluation of the Investigated Superdisintegrants

The texture analyzer-derived descriptors were transformed into SeDeM-compatible radius values and incorporated into the SeDeM-ODT profile as alternative disgregability parameters. Swelling efficiency, structural transition efficiency, and residue height were expressed as rSE, rSTE, and rRH, respectively. The resulting TA-extended SeDeM-ODT values are presented in Table 10.

The TA-extended SeDeM-ODT evaluation produced different disgregability profiles among the investigated superdisintegrants. Polyplasdone^®^ XL showed the highest TA-derived disgregability factor, with a value of 9.73, based on rSE, rSTE, and rRH values of 9.90, 10.00, and 9.29, respectively. This resulted in an IP of 0.60, an IPP of 6.09, and an IGCB of 5.91.

Polyplasdone^®^ XL-10 showed lower TA-derived radius values, with rSE, rSTE, and rRH values of 1.13, 2.91, and 6.86, respectively. Its TA-derived disgregability factor was 3.63, resulting in an IP of 0.40, an IPP of 4.08, and an IGCB of 3.96.

Vivasol showed rSE, rSTE, and rRH values of 7.66, 5.80, and 10.00, respectively. Its TA-derived disgregability factor was 7.82, with an IP of 0.40, an IPP of 4.10, and an IGCB of 3.98.

**Figure 6:**
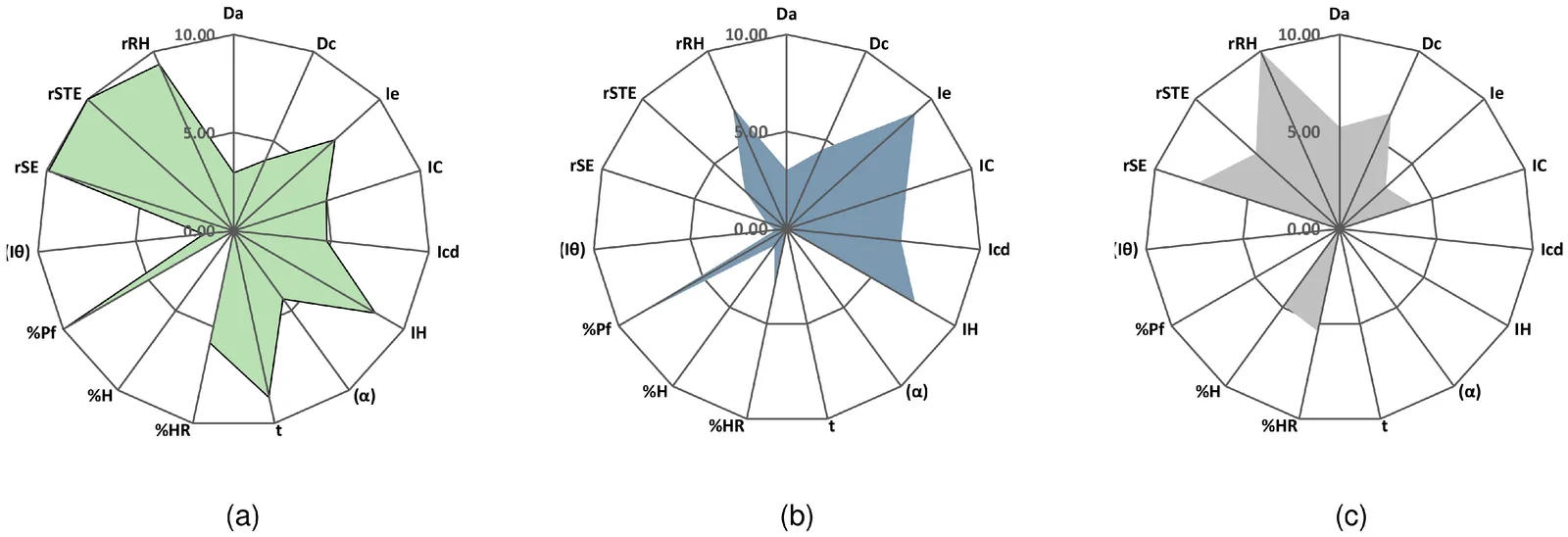
TA-extended SeDeM-ODT diagrams of the evaluated superdisintegrants: (**a**) Polyplasdone^®^ XL/crospovidone type A, (**b**) Polyplasdone^®^ XL-10/crospovidone type B, and (**c**) Vivasol/croscarmellose sodium.

The corresponding TA-extended SeDeM-ODT diagrams are shown in Figure 6.

### 3.5. Physical, Mechanical, and Disintegration Properties of Bisoprolol Fumarate ODT Formulations

Bisoprolol fumarate ODT formulations were prepared according to the central composite design and evaluated for their physical and mechanical properties. The results are presented in Table 11.

The mean tablet weight values ranged from 299.18 ± 1.54 mg to 304.09 ± 1.38 mg, indicating that the prepared tablets were close to the target weight of 300 mg. Tablet diameter values were consistent across formulations, whereas tablet thickness values showed minor variation depending on formulation composition.

Hardness values ranged from 56.23 ± 3.29 N to 197.87 ± 3.11 N. The corresponding tensile strength values ranged from 1.36 ± 0.08 MPa to 5.08 ± 0.08 MPa. Friability values ranged from 0.02% to 0.60%, and all formulations remained below the commonly applied 1% friability limit.

Pharmacopoeial disintegration times ranged from 12.24 s to 99.27 s. All formulations disintegrated within the European Pharmacopoeia requirement for orodispersible tablets [24]. These results indicate that all CCD formulations met the pharmacopoeial disintegration criterion, despite differences in mechanical strength and formulation composition.

### 3.6. In Vitro Dissolution Performance

The in vitro dissolution performance of bisoprolol fumarate ODT formulations was evaluated using corrected cumulative drug release values. The dissolution results at 5, 10, 15, and 30 min are presented in Table 12.

**Figure 7:**
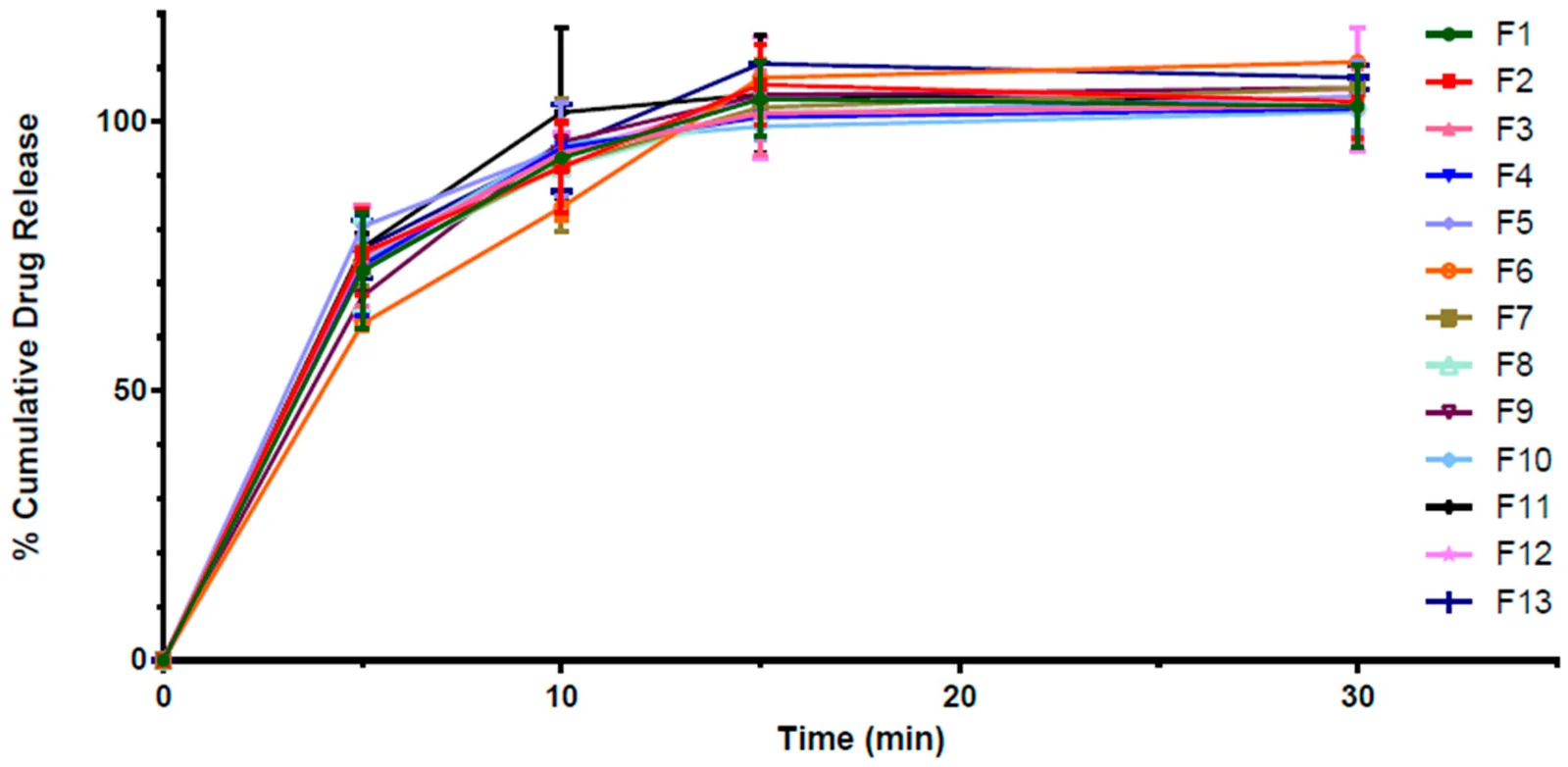
Cumulative drug release profiles of bisoprolol fumarate ODT formulations. Values are expressed as mean ± standard deviation (n = 3).

The cumulative drug release at 5 min ranged from 62.51% to 80.52%, with F5 showing the highest mean early release and F6 the lowest. F9 also showed a comparatively lower Q5 value (67.55%), whereas most of the remaining formulations released approximately 72–77% of bisoprolol fumarate at this time point. By 10 min, most formulations had released more than 90% of the drug, although F6 remained comparatively lower at 84.09%. At 15 and 30 min, the dissolution profiles largely converged, and all formulations showed near-complete drug release, indicating that the observed formulation-dependent differences were mainly confined to the initial release phase. Corrected cumulative release values were calculated from UV spectrophotometric assay results and were not truncated at 100% The cumulative dissolution profiles of the formulations are shown in Figure 7.

### 3.7. Texture Analyzer-Derived Disintegration Profiles and Descriptors of CCD Formulations

**Figure 8:**
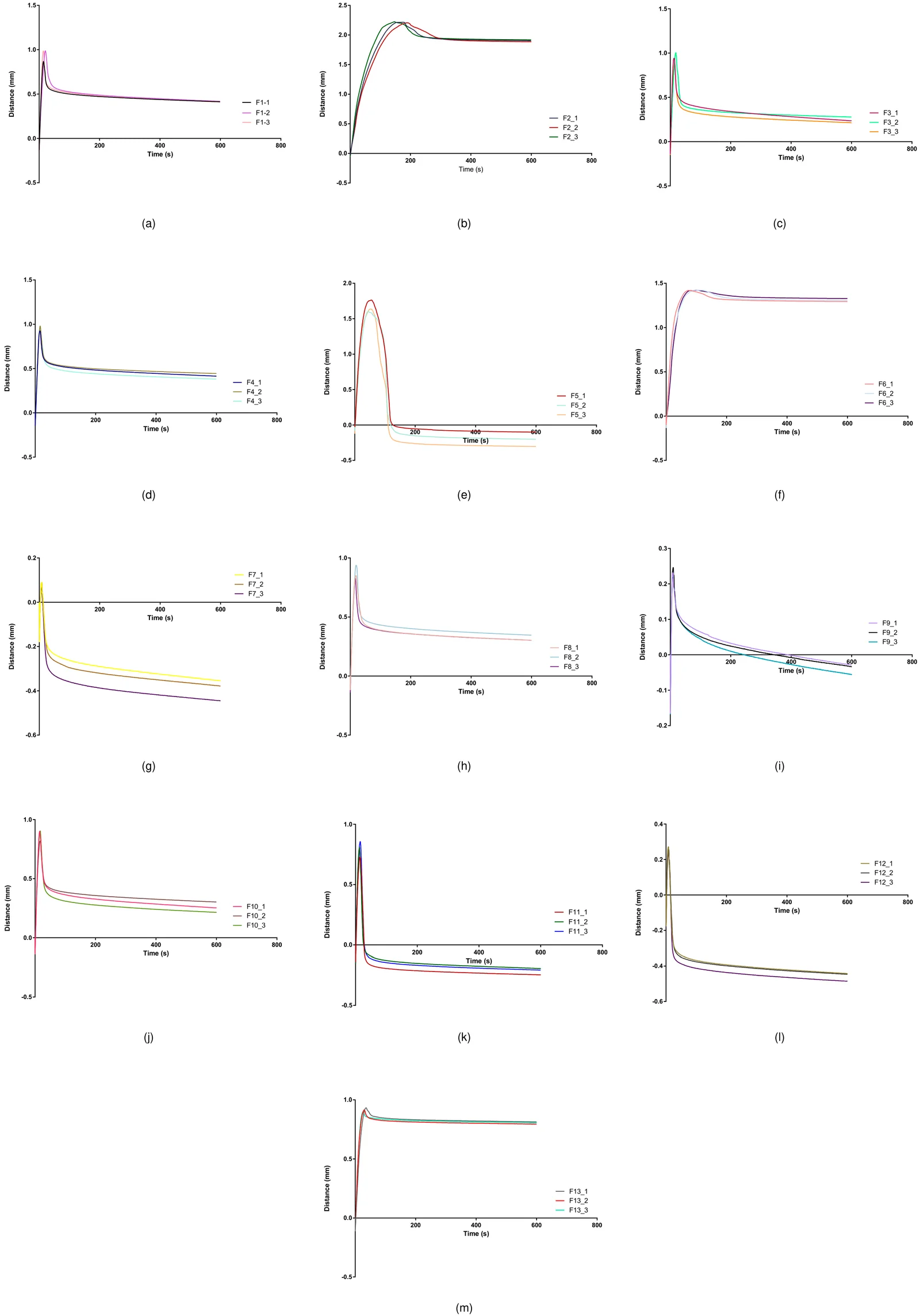
Texture analyzer distance–time profiles of bisoprolol fumarate ODT formulations prepared according to the central composite design: (**a**) F1, (**b**) F2, (**c**) F3, (**d**) F4, (**e**) F5, (**f**) F6, (**g**) F7, (**h**) F8, (**i**) F9, (**j**) F10, (**k**) F11, (**l**) F12, and (**m**) F13. Each profile represents triplicate measurements obtained under low-volume liquid controlled-force conditions.

Texture analyzer measurements were performed on all bisoprolol fumarate ODT formulations prepared according to the central composite design. The distance–time profiles were used to evaluate formulation-dependent swelling, residue height, and structural transition behavior under low-volume liquid controlled-force conditions. The representative profiles of all CCD formulations are shown in Figure 8.

The texture analyzer profiles were further processed to extract the primary profile descriptors, including maximum swelling distance (Dmax), time to maximum swelling (tA), residue height (RH), and time corresponding to the residual plateau region (tB). These values were then used to calculate swelling efficiency (SE) and structural transition efficiency (STE). The calculated descriptors are presented in Table 13.

The CCD formulations showed formulation-dependent differences in their texture analyzer-derived responses. Differences were observed in D_max_ and tA values, indicating variation in the extent and timing of the swelling response among the formulations. RH values also varied across the formulations, showing differences in the residual structural level remaining after the main transition phase. SE and STE values were subsequently used as quantitative responses for statistical model evaluation.

The center-point formulations showed comparable distance–time profiles and descriptor values, supporting the repeatability of the central design region. Formulations located at different MCC fraction and Polyplasdone^®^ XL concentration levels showed more distinct texture analyzer profiles. These differences were reflected in the extracted SE, RH, and STE values.

### 3.8. Statistical Model Evaluation of CCD Responses

The effects of MCC/LM ratio and Polyplasdone^®^ XL concentration on the selected formulation responses were evaluated using the central composite design. The statistical model summary for tensile strength, swelling efficiency, residue height, structural transition efficiency, pharmacopoeial disintegration time, and Q5 drug release percentage are presented in Table 14.

Model selection was based on sequential model testing, ANOVA, lack-of-fit analysis, adjusted R^2^, predicted R^2^, and adequate precision. A and B represent MCC/LM ratio and Polyplasdone^®^ XL concentration, respectively.

Tensile strength was fitted to a quadratic model, which was statistically significant. The MCC/LM ratio and its quadratic term were identified as significant model terms. Swelling efficiency was also fitted to a quadratic model and showed a statistically significant model response, with a non-significant lack of fit. Because significant lack of fit was observed for some responses, these models were interpreted with caution and were used primarily to describe formulation-dependent trends within the investigated design space.

Residue height and structural transition efficiency were evaluated as texture analyzer-derived responses. Structural transition efficiency was fitted to a linear model, with both MCC/LM ratio and Polyplasdone^®^ XL concentration contributing to the response. Pharmacopoeial disintegration time and dissolution rate showed non-significant overall model responses under the selected models. The selected response surface models and corresponding coded equations are presented in Table 15.

**Figure 9:**
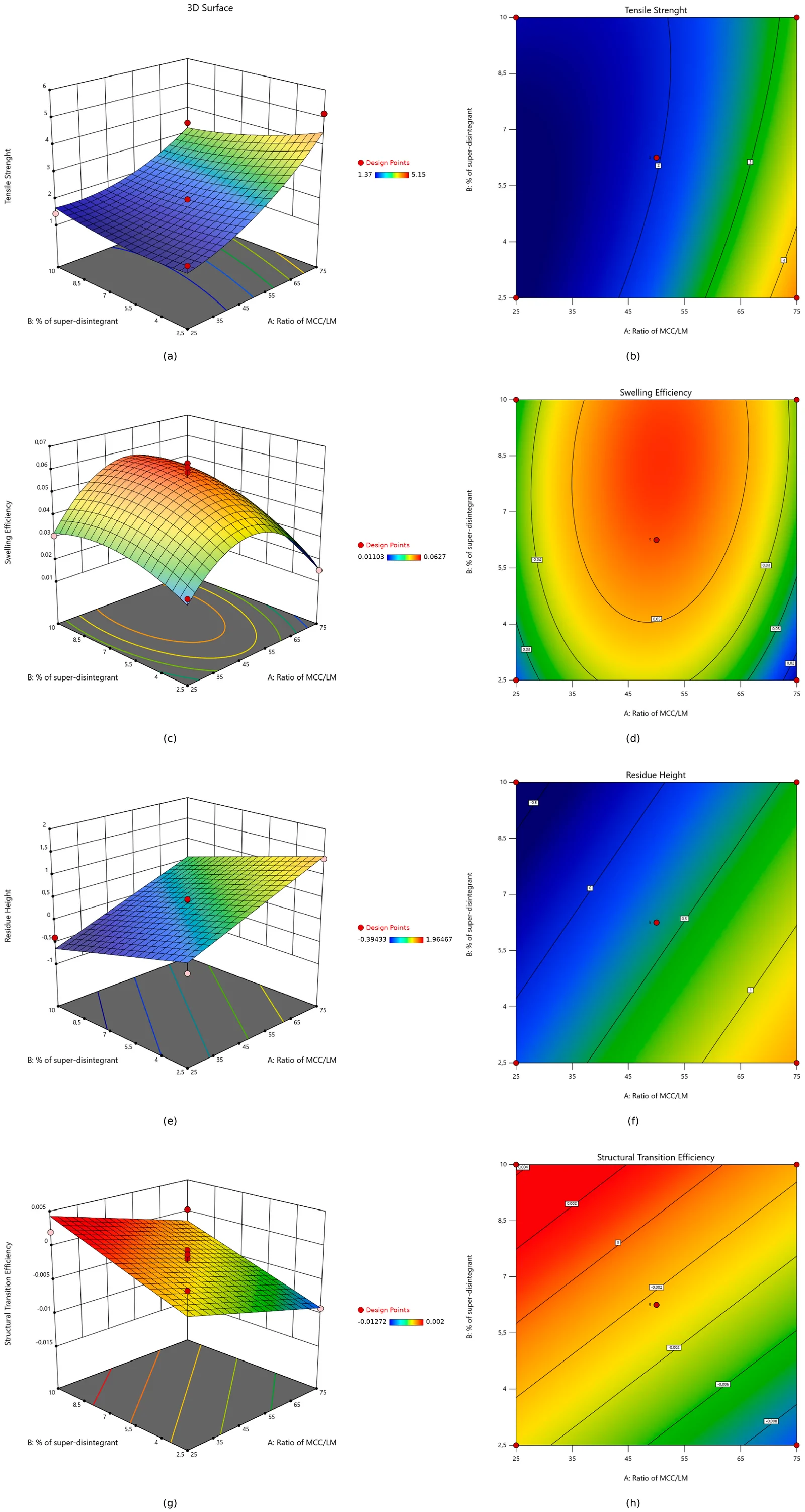
Response surface and contour plots for the investigated CCD responses: (**a**,**b**) tensile strength, (**c**,**d**) swelling efficiency, (**e**,**f**) residue height, and (**g**,**h**) structural transition efficiency.

The response surface and contour plots of the main statistically evaluated responses are shown in Figure 9.

Response surface and contour plots were generated for all evaluated responses. For responses with non-significant overall models and poor predictive performance, namely pharmacopoeial disintegration time and Q5 drug release, the plots are provided only as exploratory visualizations in Appendix A and were not used for design-space interpretation.

### 3.9. Heckel Analysis and Compression Behavior of the Investigated Excipients

Heckel analysis was performed to evalluate the compression and densification behavior of the investigated excipients. The Heckel parameters calculated from the linear region of the thickness-based Heckel plots are presented in Table 16.

The calculated Heckel slope (K), intercept (A), apparent yield pressure (Py), and coefficient of determination (R^2^) differed among the investigated excipients. The apparent yield pressure values were calculated from the reciprocal of the Heckel slope and were used to compare the densification behavior of the materials under the applied compression conditions.

**Figure 10:**
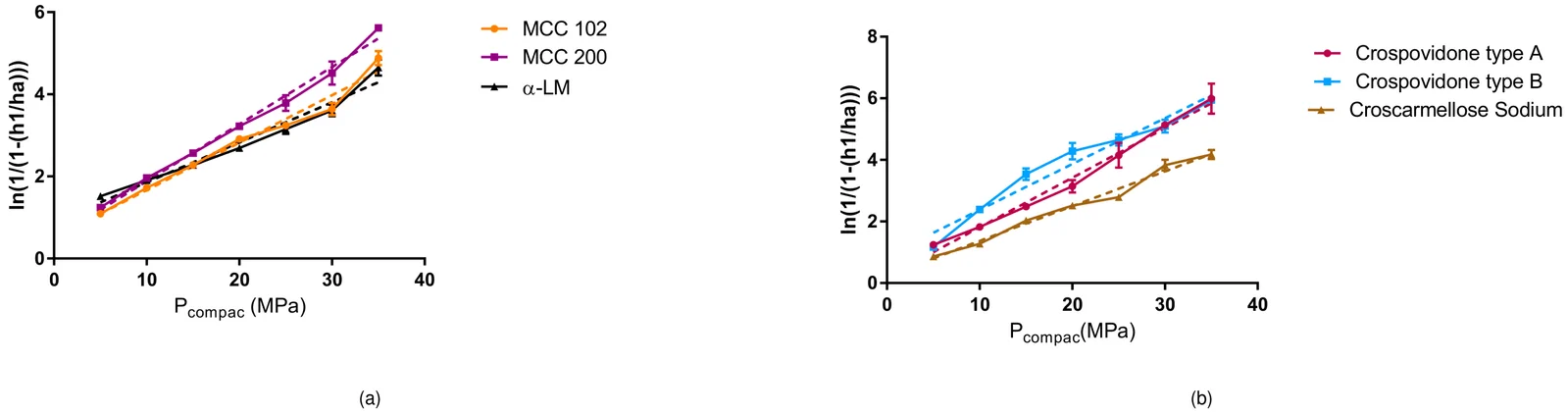
Heckel plots of the investigated excipients: (**a**) diluents and (**b**) superdisintegrant.

The Heckel plots of the investigated excipients are shown in Figure 10.

### 3.10. Comparative Kinetic Modeling of Superdisintegrants Using Texture Analyzer Profiles

Comparative kinetic modeling was performed to describe the time-dependent evolution of the texture analyzer distance–time profiles.

The swelling and structural transition phases were evaluated separately using zero-order, first-order, Higuchi, Hixson–Crowell, and Korsmeyer–Peppas models. The calculated R^2^ values for the swelling phase are presented in Table 17, and those for the structural transition phase are presented in Table 18.

The apparent yield pressure values increased in the following order: Polyplasdone^®^ XL < Polyplasdone^®^ XL-10 < Vivapur^®^ 200 < Avicel^®^ PH 102 < Vivasol^®^ < Tablettose^®^ 80.

The swelling phase was fitted using zero-order, first-order, Higuchi, Hixson–Crowell, and Korsmeyer–Peppas models. R^2^ values are presented for each formulation. The best-fit model was selected according to the highest R^2^ value, and the corresponding kinetic constant (k) is reported. Higuchi provided the best fit for F1, F2, F3, F4, F8, F10, F11, F12, and F13. Hixson–Crowell provided the best fit for F5 and F9, whereas first-order and zero-order models showed the highest R^2^ values for F6 and F7, respectively. Korsmeyer–Peppas did not provide the highest R^2^ value for any formulation during the swelling phase.

For the structural transition phase, Hixson–Crowell fitting provided the highest R^2^ values for most formulations, including F1, F3, F4, F7, F8, F10, F11, and F12. Zero-order fitting showed the highest R^2^ values for F2 and F6, whereas first-order fitting provided the best fit for F5 and F13. Higuchi provided the highest R^2^ value for F9. Korsmeyer–Peppas did not provide the highest R^2^ value for any of the evaluated formulations in the structural transition phase.

Overall, the comparative kinetic evaluation showed that the swelling phase was most frequently described by Higuchi-type profile evolution, whereas the structural transition phase was most frequently described by Hixson–Crowell-type profile evolution. These models were used only for comparative mathematical description of the texture analyzer distance–time profiles and were not intended to define a definitive disintegration or drug-release mechanism.

## 4. Discussion

### 4.1. Original SeDeM Evaluation as a Raw-Material Screening Tool and Formulation Rationale

The original twelve-parameter SeDeM Expert System was used in this study as the first screening layer to guide excipient selection for direct-compressed bisoprolol fumarate ODT development. Within this stage, the original SeDeM profile was interpreted in relation to direct-compression-relevant raw-material attributes, including dimensional characteristics, compressibility, flowability, lubricity/stability, and lubricity/dosage. Therefore, the role of the original SeDeM evaluation was not to directly predict final ODT disintegration performance, but to provide a structured basis for prioritizing excipients with suitable processing and compaction-related properties. disintegration-specific behavior was considered separately through the SeDeM-ODT framework and the subsequent texture analyzer-derived profile descriptors.

Bisoprolol fumarate showed an acceptable original SeDeM profile, with an IPP above 5 and an IGC above the conventional acceptability threshold. This indicated that the API did not present a major direct-compression limitation under the applied preformulation conditions. However, because bisoprolol fumarate accounted for only approximately 1.67% *w/w* of the final 300 mg tablet, the behavior of the final ODTs was expected to be governed primarily by the excipient matrix rather than by the API itself. Accordingly, the SeDeM evaluation of the API was mainly useful for confirming that the low-dose drug component was unlikely to impose a critical processing constraint, whereas the final mechanical, disintegration, dissolution, and texture analyzer responses were interpreted mainly in relation to the selected diluent-superdisintegrant system.

Among the evaluated diluents, Tablettose^®^ 80 showed the highest SeDeM profile. This reflected its favorable dimensional and flow-related properties and supported its role as a flow- and density-supporting component in the directly compressed matrix. However, a higher original SeDeM index for Tablettose^®^ 80 should not be interpreted as evidence that lactose alone would provide the most appropriate ODT matrix. In the present study, Tablettose^®^ 80 showed a limited cohesion-related contribution when evaluated as an individual material, suggesting that it may provide less support for compact strength under the applied conditions. This distinction is important because directly compressed ODTs require not only acceptable powder handling, but also sufficient mechanical integrity before administration.

In contrast, the MCC grades, particularly Vivapur^®^ 200, showed lower SeDeM indices than Tablettose^®^ 80, mainly because of weaker flowability-related and dimensional contributions. Nevertheless, these lower original SeDeM values were interpreted as raw-material processability limitations rather than as evidence against the formulation utility of MCC. MCC is widely used in direct compression because of its plastic deformation and bonding capacity, and its relatively weaker flow behavior can be compensated by combination with a better-flowing diluent [47]. Therefore, the SeDeM profiles supported the use of MCC not as the best single raw material according to the original index, but as a functionally important compact-forming component within a binary diluent system.

The comparison of the diluent profiles therefore provided a rationale for selecting a binary Vivapur^®^ 200/Tablettose^®^ 80 diluent system. This selection was guided by complementary excipient functionality rather than by the original SeDeM index of a single material. Vivapur^®^ 200 was included to contribute to compact formation, mechanical strength, and structural persistence, whereas Tablettose^®^ 80 was included to improve powder handling, dimensional properties, and the soluble fraction of the tablet matrix. This balance was considered particularly relevant for directly compressed ODTs, where tablet robustness and rapid liquid-mediated structural breakdown must be achieved within the same formulation.

The original SeDeM profiles of the investigated superdisintegrants were also used as part of the excipient selection strategy, but they required a distinct interpretation. Polyplasdone^®^ XL showed the most favorable original SeDeM profile among the evaluated superdisintegrants, followed by Polyplasdone^®^ XL-10 and Vivasol^®^. This ranking indicated that Polyplasdone^®^ XL had comparatively better direct-compression-related raw-material suitability within the tested superdisintegrant group. However, the original twelve-parameter SeDeM system does not contain disgregability parameters and therefore cannot, by itself, describe the functional disintegration mechanism of superdisintegrants. For this reason, the original SeDeM results were used to guide candidate prioritization, not to make a final claim regarding ODT disintegration performance.

The selection of Polyplasdone^®^ XL for the subsequent design-based formulation study was therefore based on a sequential and integrated rationale. First, the original SeDeM evaluation identified Polyplasdone^®^ XL as the most favorable superdisintegrant candidate in terms of direct-compression-related raw-material properties. Second, this selection was further examined using conventional SeDeM-ODT and texture analyzer-derived evaluations, as discussed in the following sections. Thus, Polyplasdone^®^ XL was not selected solely on the basis of the original SeDeM profile, but through the convergence of raw-material processability and subsequent disintegration-related functional assessment.

Similarly, the Vivapur^®^ 200/Tablettose^®^ 80 diluent system was selected because the two materials offered complementary functions within the directly compressed ODT matrix. Tablettose^®^ 80 contributed favorable flow and soluble matrix characteristics, whereas Vivapur^®^ 200 was expected to support compact formation and mechanical robustness. This combination provided a rational formulation platform for evaluating the effects of MCC/lactose balance and Polyplasdone^®^ XL concentration during the subsequent central composite design.

Overall, the original SeDeM evaluation served as a SeDeM-guided excipient selection and formulation-rationale tool. It established the raw-material basis for selecting the binary diluent system and prioritizing the superdisintegrant candidate, while recognizing that final ODT performance requires additional formulation-level evaluation. The subsequent conventional SeDeM-ODT assessment, texture analyzer-derived descriptors, CCD-based formulation modeling, pharmacopoeial disintegration testing, dissolution testing, and Heckel analysis were therefore used as complementary layers to evaluate whether the selected excipient system could provide mechanical robustness and profile-level discrimination within a pharmacopoeially compliant ODT design space.

### 4.2. Conventional SeDeM-ODT Evaluation and the Limitation of Endpoint-Based Disgregability Parameters

The investigated superdisintegrants were further evaluated using the conventional SeDeM-ODT approach, in which the original twelve-parameter SeDeM profile is extended by three endpoint-based Disgregability parameters: effervescence/dispersion time (DE), disintegration time with disk (DCD), and disintegration time without disk (DSD). Accordingly, the original index used in this section was the Index of Good Compressibility and Bucodispersibility (IGCB), rather than the original Index of Good Compressibility (IGC). This distinction is important because the conventional SeDeM-ODT profile does not only describe direct-compression suitability; it also incorporates an endpoint-based assessment of disgregability relevant to ODT development.

In the present study, the conventional SeDeM-ODT evaluation ranked Polyplasdone^®^ XL as the most favorable superdisintegrant among the tested materials, followed by Polyplasdone^®^ XL-10 and Vivasol^®^ in terms of original IGCB values. However, none of the pure superdisintegrant compacts reached the conventional original acceptability threshold. This finding should be interpreted at the level of pure compact evaluation and should not be considered a direct prediction of finished ODT performance. Superdisintegrants are not normally used as isolated compacted materials; rather, they are incorporated at relatively low concentrations into multicomponent tablet matrices, where their functionality depends on diluent composition, porosity, compression pressure, tablet microstructure, and liquid penetration pathways.

The low conventional SeDeM-ODT indices observed for the pure superdisintegrants therefore indicate limitations under the specific endpoint-based test conditions, rather than poor functional suitability for ODT formulation. This distinction is particularly important for polymeric superdisintegrants such as crospovidone and croscarmellose sodium, which may hydrate, swell, deform, or remain as cohesive hydrated masses when tested as pure compacts. Such behavior can interfere with endpoint-based interpretation, especially when complete dispersion or passage through the test apparatus is required as the operational criterion.

Among the conventional disgregability parameters, DE provided limited useful differentiation for the present materials. This is not unexpected, because the investigated superdisintegrants are not effervescent materials and their functional contribution is mainly associated with liquid uptake, swelling, wicking, strain recovery, and disruption of the tablet matrix. Therefore, failure to obtain a favorable DE radius value should not be interpreted as absence of water interaction or disintegration functionality. Rather, it suggests that DE may be a limited descriptor for non-effervescent, swelling-dominated polymeric superdisintegrants when they are evaluated as pure compacts.

A similar limitation was observed for the disk-free disintegration condition. Under disk-free testing, hydrated polymer compacts may swell, float, deform, or retain a coherent gel-like or porous structure rather than reaching a clear endpoint. This behavior reflects the physical response of the pure hydrated compact, but it does not necessarily represent the behavior of the same material when dispersed within a porous tablet matrix containing soluble and compactible diluents. In this sense, swelling and disintegration should not be treated as equivalent events. A material may exhibit extensive hydration or swelling while retaining residual structure, whereas a complete formulation may disintegrate effectively due to the combined effects of superdisintegrant action, soluble matrix components, and tablet porosity.

The disintegration test performed with disk provided comparatively more differentiation among the investigated superdisintegrants. The presence of the disk may impose mechanical restraint and improve contact between the compact and the aqueous medium, thereby producing a more defined endpoint for swelling polymer compacts. Nevertheless, the overall conventional Disgregability factor remained low because the endpoint-based parameters did not capture the dynamic sequence of swelling, weakening, collapse, and residual structure formation.

These findings highlight the main limitation of the conventional SeDeM-ODT disgregability factor in the context of the present study. Conventional DE, DCD, and DSD parameters are useful for providing a standardized endpoint-based reference, but they offer limited information about the time-dependent structural changes that occur before the final endpoint is reached. They do not distinguish whether a material undergoes rapid swelling followed by collapse, slow swelling with persistent structure, or limited swelling with rapid matrix loss. This limitation becomes particularly relevant when materials or formulations exhibit broadly acceptable endpoint performance yet differ in their underlying structural pathways.

Therefore, the conventional SeDeM-ODT results were not considered sufficient as the sole basis for mechanistic superdisintegrant selection. Instead, they were used as a reference endpoint-based evaluation that justified the need for an additional profile-based approach. The subsequent texture analyzer-derived descriptors were developed to complement, rather than replace, the conventional SeDeM-ODT parameters by quantifying swelling efficiency, residual structure, and structural transition behavior under controlled low-volume liquid conditions.

### 4.3. Texture Analyzer-Derived Descriptors as a Profile-Based Extension of SeDeM-ODT

The conventional SeDeM-ODT disgregability factor provided an endpoint-based reference for the investigated superdisintegrants, but it did not describe how the materials behaved before reaching, or failing to reach, the final endpoint. For this reason, texture analyzer-derived descriptors were incorporated as a profile-based extension of the SeDeM-ODT framework. The purpose of this extension was not to replace the conventional DE, DCD, and DSD parameters, but to complement them with quantitative information obtained from the continuous distance–time response of the compact during exposure to a limited aqueous volume under controlled force.

This distinction is important because disintegration is not a single event. It involves a sequence of liquid penetration, swelling, matrix weakening, structural rearrangement, collapse, and residual structure formation. Conventional endpoint tests describe the final observable outcome of this sequence, whereas texture analyzer profiles allow different regions of the same sequence to be examined separately. In the present study, this was particularly useful because the investigated superdisintegrants showed different combinations of swelling rate, maximum swelling, residue height, and time to residual plateau. These differences could not be fully represented by a single endpoint disintegration or dispersion value.

Three descriptors were therefore selected from the texture analyzer profiles: swelling efficiency (SE), residue height (RH), and structural transition efficiency (STE). Each descriptor was intended to represent a different aspect of the profile. SE was used to describe the rate-normalized development of swelling, calculated from the maximum swelling distance and the time required to reach it. RH was used to describe the residual structural level remaining after the main transition phase. STE was used to describe the transition toward residual structure loss in relation to the time required to reach the residual plateau. Together, these descriptors provide a more multidimensional description of disintegration-related behavior than endpoint-based parameters alone.

SE was especially useful for distinguishing materials that showed comparable swelling amplitudes but different swelling rates. Polyplasdone^®^ XL and Vivasol^®^ both exhibited marked upward displacement, indicating strong swelling behavior under the applied low-volume liquid conditions. However, Polyplasdone^®^ XL reached its maximum swelling point more rapidly, resulting in the highest SE value among the investigated superdisintegrants. Vivasol^®^ showed a similar or slightly higher maximum swelling distance, but the longer time required to reach this maximum reduced its SE value. This distinction is relevant because rapid initial swelling can contribute to early matrix disruption in ODTs, whereas a slower swelling response may lead to a different disintegration pathway, even when the maximum swelling extent is high.

Polyplasdone^®^ XL-10 showed a clearly different swelling profile. Its lower maximum swelling distance and much longer time to maximum swelling resulted in a markedly lower SE value. This behavior is consistent with a slower development of swelling under the applied conditions. Importantly, this type of difference is not adequately captured by conventional endpoint-based disgregability testing. The material may eventually hydrate or deform, but the time course of this response can be critical for understanding its contribution to rapid ODT disintegration. Therefore, SE added useful information by separating swelling extent from swelling rate.

RH provided a second, and arguably the most directly interpretable, dimension of the texture analyzer response. Unlike SE, which describes the early swelling phase, RH describes the structural state remaining after the main transition phase. Negative RH values indicate collapse or downward displacement relative to the initial reference position, whereas positive RH values indicate persistent structure after liquid exposure. This descriptor is important because swelling alone is not equivalent to disintegration. A compact may swell strongly and still retain a coherent hydrated structure; conversely, a material may show limited upward swelling but undergo substantial matrix loss or collapse. Because RH was defined relative to the initial distance level, negative RH values indicated that the texture analyzer signal moved below the initial reference position after the main transition phase. In this operational sense, negative RH was interpreted as structural collapse or downward displacement under the applied low-volume liquid controlled-force conditions. However, the distance–time profile alone cannot distinguish whether this response resulted primarily from matrix collapse, dissolution or erosion of soluble components, fragmentation, or force-induced structural failure. Therefore, collapse-related interpretations in this study should be understood as profile-based operational interpretations rather than direct proof of a single physical mechanism.

The behavior of the reference excipients supported this interpretation. Tablettose^®^ 80 did not show a measurable upward swelling maximum under the applied test conditions, which resulted in SE being assigned as zero. However, it showed a negative RH value, indicating downward displacement or loss of structure after liquid exposure. This profile is consistent with the role of lactose as a soluble diluent that does not form a persistent swollen network under low-volume liquid controlled-force conditions. In contrast, Vivapur^®^ 200 showed prolonged swelling and a positive RH value, indicating a persistent hydrated structure. This behavior is consistent with the structure-forming and water-interacting characteristics of MCC. The contrasting responses of Tablettose^®^ 80 and Vivapur^®^ 200 provided useful reference points for interpreting the behavior of the superdisintegrants and, later, the CCD formulations.

Among the superdisintegrants, Vivasol^®^ showed the most negative RH value, indicating the greatest collapse-related downward displacement of the texture analyzer signal after liquid exposure. Polyplasdone^®^ XL also showed a clearly negative RH value, whereas Polyplasdone^®^ XL-10 showed a less negative RH and a much longer time to residual plateau. These differences suggest that the three superdisintegrants did not simply differ in the extent of disintegration, but in the profile pathway through which swelling, structural transition, and residual persistence occurred. Vivasol^®^ was characterized by pronounced collapse-related signal displacement; Polyplasdone^®^ XL combined rapid swelling with an efficient transition toward structural loss; and Polyplasdone^®^ XL-10 showed slower and more persistent behavior under the applied conditions.

The interpretation of STE requires more caution. STE was calculated from RH and tB; therefore, it is mathematically linked to residue height and should not be considered a fully independent mechanistic endpoint. Its value lies in expressing the transition toward structural loss over time. For example, a material with a negative RH reached after a shorter tB will show a higher positive STE than a material that reaches a similar residual state more slowly. In this sense, STE complements RH by incorporating the time dimension of the transition phase. However, because STE depends directly on RH, it should be interpreted as a time-normalized companion descriptor rather than as a separate proof of disintegration mechanism.

This distinction was important in the present results. Polyplasdone^®^ XL showed the highest STE value among the investigated superdisintegrants, reflecting the combination of a strongly negative RH and a relatively shorter time to residual plateau. Vivasol^®^ showed a more negative RH than Polyplasdone^®^ XL, but because it reached the residual plateau later, its STE value was lower. This indicates that Vivasol^®^ showed substantial collapse-related downward displacement, but the transition occurred over a longer time scale than that observed for Polyplasdone^®^ XL. Polyplasdone^®^ XL-10 showed both slower swelling development and lower transition efficiency. Therefore, the combined interpretation of SE, RH, and STE provides a clearer description of the superdisintegrant profiles than any single descriptor alone.

The transformation of SE, RH, and STE into SeDeM-compatible radius values enabled the incorporation of these profile-derived descriptors into the familiar 0–10 SeDeM-ODT scale. The normalization limits for SE, STE, and RH were empirically derived from the experimentally observed extremes within the investigated material set. Specifically, the lower limit for SE (0) corresponds to the absence of measurable swelling in α-lactose monohydrate, whereas the upper limit (0.044 mm/s) corresponds to the rapid swelling response of Vivasol^®^. This approach ensures that the transformed radius values (rSE, rSTE, rRH) are grounded in the actual formulation-relevant behavior of the tested materials, rather than arbitrary theoretical boundaries. Nevertheless, these limits are dataset-specific and should be validated with a broader excipient library before generalization. This was useful for comparing the TA-derived disgregability profile with the conventional endpoint-based SeDeM-ODT profile. In the TA-extended SeDeM-ODT evaluation, Polyplasdone^®^ XL showed the most favorable overall disgregability profile, with high rSE, rSTE, and rRH values. This indicated rapid swelling, efficient transition toward structural loss, and low residual structural persistence under the applied low-volume liquid conditions. Vivasol^®^ also showed a strong TA-derived disgregability contribution, especially through rRH, but its lower rSE and rSTE values indicated a different profile pathway. Polyplasdone^®^ XL-10 exhibited the weakest TA-derived disgregability profile, primarily due to its slow swelling kinetics and lower transition efficiency.

The comparison between conventional and TA-extended SeDeM-ODT profiles illustrates the value of the proposed extension. The conventional SeDeM-ODT approach ranked the superdisintegrants using endpoint-based parameters, but the disgregability factor remained low and did not explain the underlying profile differences. The TA-extended approach retained the same SeDeM-compatible structure while adding information on swelling rate, residual structure, and time-normalized structural transition. Therefore, the TA-derived descriptors did not simply change the numerical ranking of the materials; they improved the interpretability of why the materials behaved differently under liquid exposure.

This point is particularly relevant for polymeric superdisintegrants. Crospovidone and croscarmellose sodium are not expected to behave like effervescent or completely soluble disintegration aids. Their performance depends on liquid uptake, swelling pressure, strain recovery, matrix disruption, particle morphology, and interaction with the surrounding tablet network [48,49]. Endpoint-based tests may not distinguish these contributions when the material forms a hydrated cohesive mass or when the final endpoint is difficult to define. Texture analyzer profiles provide a way to observe these intermediate structural events quantitatively, even when complete disintegration is not captured by the conventional endpoint definition.

The inclusion of non-superdisintegrant reference excipients also strengthened the interpretation of the TA-derived descriptors. The lactose reference represented a soluble, low-structure-persistence behavior, whereas MCC represented a swelling-prone and structure-retaining behavior. By positioning the superdisintegrants between or beyond these reference behaviors, the proposed descriptors gained practical interpretability. For example, high SE alone was not sufficient to define a favorable ODT-related response if residual structure persisted. Similarly, negative RH alone did not describe whether structural loss occurred rapidly or slowly. The combined use of SE, RH, and STE therefore provided a more complete profile-based interpretation. The ≤1% variation over 30 s criterion was selected as a stringent operational definition of a sustained residual plateau; although alternative thresholds could shift Point B in gradually stabilizing profiles, the same criterion was applied uniformly throughout the study.

Nevertheless, the TA-extended SeDeM-ODT approach should be interpreted as an empirical and comparative extension rather than as a finalized universal scoring system. The normalization limits used for rSE, rSTE, and rRH were derived from the experimental range of the materials included in this study, including the three superdisintegrants and two reference excipients. Therefore, these limits are practical boundaries for the present dataset and should not yet be considered general acceptance criteria for all ODT excipients. Broader validation across additional superdisintegrant types, diluent systems, compression pressures, and tablet geometries would be required before universal radius limits could be proposed.

A further limitation is that the texture analyzer method was performed under a specific low-volume liquid, controlled-force condition. This condition was selected to provide discriminatory structural information, but it does not reproduce the full complexity of oral disintegration, where saliva volume, tongue pressure, patient variability, and residence time may differ. Therefore, the descriptors should be considered formulation-development tools that provide comparative structural insight, not direct predictors of in vivo mouthfeel or clinical performance. This limitation does not reduce their usefulness; rather, it defines the appropriate scope of their interpretation.

Overall, the texture analyzer-derived extension of SeDeM-ODT provided a more informative description of superdisintegrant behavior than conventional endpoint-based Disgregability testing alone. SE described the rate of swelling development, RH described the residual structural state, and STE described the time-normalized transition toward structural loss. The combined interpretation of these descriptors supported the selection of Polyplasdone^®^ XL for the CCD formulations. This selection was not based solely on the original SeDeM or conventional SeDeM-ODT ranking, but on the broader evidence that Polyplasdone^®^ XL provided the most favorable profile balance of rapid swelling, low residual structural persistence, and efficient structural transition under the applied low-volume liquid conditions.

### 4.4. Formulation-Level Discrimination Within a Pharmacopoeially Relevant ODT Design Space

The CCD formulations were developed to evaluate whether the proposed texture analyzer-derived descriptors could provide additional discriminatory information within a formulation space that already showed acceptable conventional ODT performance. This point is central to the interpretation of the formulation results. The objective of the CCD stage was not merely to obtain bisoprolol fumarate ODTs that disintegrated rapidly and released the drug efficiently, but to determine whether formulation variables could still be differentiated when conventional endpoint responses were no longer sufficiently sensitive. In other words, the CCD formulations provided a practical test case for evaluating whether low-volume liquid texture analyzer profiling could reveal structural differences that remained hidden behind broadly acceptable pharmacopoeial disintegration and rapid dissolution behavior.

All formulations fulfilled the pharmacopoeial disintegration requirement for ODTs, with disintegration times remaining well below the 3 min limit. This confirmed that the selected excipient system was suitable for the preparation of rapidly disintegrating bisoprolol fumarate tablets by direct compression. The shortest disintegration times were observed in formulations containing higher lactose contribution and/or higher Polyplasdone^®^ XL levels, whereas the longest disintegration time was obtained for the formulation containing a high MCC fraction and a low superdisintegrant level. This trend is consistent with the expected formulation roles of the excipients: lactose contributes a soluble and relatively less structure-retaining matrix fraction, whereas MCC contributes compact strength and hydrated structural persistence; Polyplasdone^®^ XL facilitates liquid uptake and matrix disruption. However, despite these numerical differences, the pharmacopoeial disintegration response remained within a narrow compliant range relative to the official ODT limit. Therefore, although the test confirmed acceptability, it did not fully describe the structural pathway by which the formulations reached disintegration.

When the conventional tablet properties were considered together, the observed formulation-dependent differences were primarily associated with the MCC/LM balance and Polyplasdone^®^ XL concentration. MCC-rich formulations generally exhibited higher hardness and tensile strength and, descriptively, lower friability, consistent with the formation of a more coherent compact matrix. In contrast, lactose-rich formulations showed lower mechanical strength and higher friability values, although all formulations remained below the 1% friability limit. Because friability was not included as a response in the CCD model, this pattern was interpreted descriptively rather than predictively. Despite the wide range of mechanical strength values, rapid disintegration was maintained through the combined contribution of the soluble, less structure-retaining lactose fraction and Polyplasdone^®^ XL-mediated liquid uptake and matrix disruption. Accordingly, the longest disintegration time was observed for the formulation containing a high MCC fraction and a low Polyplasdone^®^ XL level, whereas shorter disintegration times were generally observed in formulations with a higher lactose contribution and/or higher Polyplasdone^®^ XL concentration.

This limitation was also reflected in the statistical model evaluation. Pharmacopoeial disintegration time did not produce a statistically significant predictive model within the investigated design space. This finding should not be interpreted as a failure of the formulations, but rather as evidence that the selected formulation domain had already entered a rapidly disintegrating region. Once all formulations disintegrate far below the pharmacopoeial threshold, the endpoint disintegration test may reach a practical ceiling effect and lose discriminatory sensitivity. Under such conditions, small numerical differences in disintegration time may not adequately represent differences in swelling kinetics, matrix weakening, residual structure, or collapse behavior. This explains why an additional profile-based method was needed to characterize formulation-level behavior more deeply.

The dissolution results should be interpreted in a similar but more carefully qualified manner. Under the modified comparative dissolution method used in this study, all formulations showed rapid bisoprolol fumarate release and exceeded the 75% Q reference value before the pharmacopoeial 45 min time point. This supports the conclusion that the formulations provided rapid drug release, which is desirable for an ODT system containing a highly soluble, low-dose model drug. However, the dissolution method used here was intentionally modified from the official bisoprolol tablet monograph conditions. In particular, the medium volume was reduced to 500 mL and UV-based quantification was used to obtain analytically suitable absorbance values for the low-dose formulation. Therefore, the dissolution data should be considered comparative rapid-release data obtained with reference to the pharmacopoeial Q criterion, rather than a full monograph-equivalent dissolution compliance claim.

Within this modified dissolution method, the profiles were broadly similar among the formulations. It should be emphasized that the dissolution method was modified from the official monograph (500 mL instead of 900 mL) to enable reliable UV quantification at the low drug dose. While this prevents formal pharmacopoeial compliance claims, the results unequivocally confirm rapid drug release (>75% at 45 min) under the tested conditions. Most formulations released a high fraction of bisoprolol fumarate during the early sampling period, and near-complete release was observed by the later sampling points. The Q5 response showed some numerical variation, but the corresponding statistical model had limited predictive value. This suggests that early dissolution was not sufficiently sensitive to the investigated formulation variables within the selected design space. Several factors may explain this observation. First, bisoprolol fumarate was present at a low drug load, corresponding to approximately 1.67% *w/w* of the tablet mass, so the overall release behavior was strongly influenced by rapid tablet wetting and matrix breakdown rather than by a high drug load. Second, the formulations were all designed as rapidly disintegrating systems, and once rapid matrix opening occurred, the soluble drug could be released efficiently across the formulation set. Third, the dissolution test was conducted under an excess aqueous environment compared with the low-volume liquid conditions relevant to the oral cavity. As a result, formulation-dependent differences in early swelling or residual structure may have been partially masked by the availability of dissolution medium.

In contrast, the texture analyzer-derived responses retained formulation-level resolution under low-volume liquid controlled-force conditions. The distance–time profiles showed clear differences in the extent and rate of swelling, the residual structural level after the main transition phase, and the time-dependent tendency toward structural loss. These differences were captured by SE, RH, and STE, which responded more clearly to the MCC/LM ratio and Polyplasdone^®^ XL concentration than pharmacopoeial disintegration time or Q5 release. This supports the central hypothesis of the study: profile-derived descriptors can provide additional formulation discrimination in an ODT design space where conventional endpoint tests confirm acceptability but do not fully explain structural behavior.

Swelling efficiency was the most statistically robust texture analyzer-derived response. The significant quadratic model and non-significant lack of fit indicate that SE captured a systematic relationship between formulation composition and rate-normalized swelling behavior. The response surface suggested that intermediate regions of the design space favored more efficient swelling. This curvature is captured by the negative A^2^ and B^2^ terms in the coded quadratic equation for SE, indicating an optimum range rather than a simple linear increase. This observation is formulation-relevant because ODT performance depends not only on the maximum extent of liquid uptake or swelling, but also on how quickly the structure responds after contact with a limited volume of fluid. A formulation that swells extensively but slowly may not provide the same disintegration experience as a formulation that develops swelling rapidly and then transitions toward matrix breakdown. Therefore, SE provided information that could not be obtained from endpoint disintegration time alone.

The influence of the MCC/LM ratio on SE also reflects the balance between structure formation and structural weakening. MCC-rich formulations tended to show stronger and more persistent compact structure, which can support mechanical robustness but may also slow the apparent development of swelling or maintain a more coherent hydrated network. Lactose-rich formulations, on the other hand, may facilitate faster matrix weakening because of the soluble diluent fraction, but excessive reduction of the structure-forming MCC fraction may limit the development of an organized swelling response. Therefore, the observed SE behavior suggests that the most favorable swelling response did not arise from the extreme dominance of one diluent, but from a balanced matrix composition in which liquid penetration, swelling, and structural weakening could occur in a coordinated manner.

Residue height provided a second and highly interpretable dimension of formulation behavior. While SE describes the rate of swelling development, RH describes the structural state remaining after the main transition phase. Positive RH values indicate that a measurable structure persisted above the initial reference level, whereas negative RH values indicate collapse or downward displacement relative to the initial position. This distinction is important because two formulations may both satisfy the pharmacopoeial disintegration endpoint while leaving different levels of hydrated residual structure under low-volume liquid conditions. Such residual structure may be relevant for understanding the physical pathway of disintegration, even when it does not prevent the formulation from meeting the official endpoint requirement.

The RH results showed a clear formulation trend. MCC-rich formulations tended to retain higher residue height, whereas lactose-rich formulations showed lower or negative RH values. As reflected by the positive coefficient of A and negative coefficient of B in the linear RH equation, the MCC fraction promotes structural persistence, while the superdisintegrant promotes collapse. This is consistent with the expected structural contribution of MCC as a plastically deforming, water-interacting, structure-forming diluent. MCC can contribute to compact strength and maintain a hydrated, porous network during exposure to liquid. In contrast, the lactose-rich matrix contains a greater soluble fraction, which may promote matrix weakening, erosion, or collapse under the applied low-volume liquid force conditions. Therefore, RH served as a direct indicator of the balance between hydrated structural persistence and collapse within the formulation matrix.

However, the RH model should be interpreted with appropriate caution. Although the MCC/LM ratio and Polyplasdone^®^ XL concentration showed significant effects, the significant lack of fit indicates that the selected model did not fully capture all sources of variation in the RH response. This is not surprising because several coupled factors, including tablet porosity, compact tensile strength, liquid penetration pathway, local distribution of superdisintegrant particles, and the balance between swelling pressure and matrix weakening likely influence residue height. Therefore, RH should be considered a descriptive and mechanistically informative response within the present formulation space, rather than a fully predictive universal model.

Structural transition efficiency provided a complementary interpretation of the same structural transition process. Because STE is calculated from RH and the time required to reach the residual plateau, it describes the transition toward residual structure loss in a time-normalized manner. Formulations with negative RH values yielded positive STE values, indicating a more efficient transition toward collapse or loss of residual structure. This is mathematically consistent with the coded equation, where STE is driven by the opposing effects of A and B. In contrast, formulations with persistent positive RH values generated negative STE values, reflecting retained structure rather than collapse. This makes STE useful for comparing not only whether residual structure remained, but also how rapidly the formulation reached that residual state.

Nevertheless, STE should not be overinterpreted as an independent mechanistic endpoint. Since it is derived from RH and tB, it is mathematically linked to the residue-height descriptor. Its value lies in combining the direction of structural change with the time required to reach the residual plateau. Therefore, in the present study, STE was interpreted as a complementary time-normalized descriptor, whereas RH was considered the more direct indicator of residual structural persistence or collapse. This interpretation avoids overstating STE’s independence while preserving its usefulness as a profile-derived response.

The combined interpretation of SE, RH, and STE indicates that the texture analyzer-derived descriptors capture distinct aspects of ODT structural behavior. SE reflects the rate of swelling development, RH reflects the final residual structural level, and STE reflects the time-normalized transition toward structural loss. This multidimensional interpretation is more informative than relying on a single endpoint disintegration time. For example, an MCC-rich formulation may show acceptable pharmacopoeial disintegration but retain a higher residual structure under low-volume liquid texture analysis. Conversely, a lactose-rich formulation may disintegrate rapidly and show a lower residue height, indicating a stronger tendency toward collapse or matrix loss. Both formulations may be acceptable by conventional criteria, but their structural pathways differ.

This distinction is particularly relevant to ODT formulation development because the oral cavity is a low-volume liquid, mechanically dynamic environment, unlike the large-volume aqueous environment used in compendial disintegration or dissolution tests. Although the texture analyzer method used in this study does not directly reproduce the oral cavity, it provides a controlled, low-volume liquid condition under which formulation-dependent swelling and structural persistence can be compared. Therefore, the TA-derived descriptors should be viewed as formulation-development tools that provide additional structural insight, rather than as replacements for official pharmacopoeial tests.

Taken together, the formulation-level results demonstrate a layered interpretation of ODT performance. Pharmacopoeial disintegration confirmed that all formulations met the required ODT endpoint. Modified comparative dissolution testing confirmed rapid bisoprolol fumarate release with reference to the 75% Q criterion. However, these conventional responses had limited ability to discriminate between formulations within the investigated compliant design space. Texture analyzer-derived descriptors provided additional resolution by revealing differences in swelling efficiency, residual structure, and structural transition behavior. Therefore, the CCD results support the main premise of this study: low-volume liquid texture analyzer-derived descriptors complement pharmacopoeial and dissolution endpoints by providing profile-level structural information that is particularly useful when conventional endpoints are already satisfied.

### 4.5. Compression Behavior and Structural Persistence: Supportive Interpretation from Heckel Analysis

Heckel analysis was used as a supportive material-level tool to contextualize the texture analyzer-derived structural responses. The aim was not to establish a direct causal relationship between compression behavior and disintegration behavior, but to determine whether the apparent densification tendencies of individual excipients were broadly consistent with the structural persistence or collapse-related responses observed during low-volume liquid texture analyzer testing. Because Heckel analysis was performed on pure excipient compacts, whereas the CCD formulations were multicomponent tablets, the results were interpreted comparatively and cautiously.

The apparent yield pressure values followed the order Polyplasdone^®^ XL < Polyplasdone^®^ XL-10 < Vivapur^®^ 200 < Avicel^®^ PH 102 < Vivasol^®^ < Tablettose^®^ 80. This ranking indicated differences in apparent densification behavior under the applied compression conditions. Lower apparent Py values were considered consistent with a greater tendency toward plastic densification, whereas higher values indicated greater resistance to densification and/or a more fragmentation-dominant response. However, because true density was not measured and out-of-die thickness-based apparent density was used, these values should be regarded as comparative apparent parameters rather than absolute material constants.

The diluent comparison is particularly relevant for interpreting the formulation-level texture analyzer responses. MCC-rich formulations showed higher tensile strength and greater residue height under low-volume liquid texture analyzer testing. This behavior can be interpreted as the result of a more coherent compact structure formed by MCC-rich matrices. The lower Py of MCC grades compared to lactose is consistent with the higher tensile strength and residue height observed in MCC-rich formulations. However, this relationship is associative rather than causative, as Heckel analysis reflects dry compression behavior, whereas TA profiles reflect post-compaction liquid-mediated structural changes. MCC is known to contribute to tablet strength through plastic deformation and interparticulate bonding, and the relatively low apparent yield pressure of Vivapur^®^ 200 in the present study supports its ability to densify and form a persistent compact network. Under limited-fluid conditions, such a network may resist complete structural collapse and remain detectable as positive or higher residue height in the distance–time profile.

In contrast, lactose-rich formulations tended to show lower residue height or negative RH values, indicating greater structural collapse or downward displacement during the texture analyzer test. Tablettose^®^ 80 showed the highest apparent yield pressure in the Heckel analysis, suggesting greater resistance to plastic densification and a comparatively lower tendency to form a persistent plastically bonded structure under compression. In a formulation matrix, the soluble nature of lactose may further contribute to liquid-mediated weakening and loss of structure. Therefore, the lower residue height observed in lactose-rich formulations is consistent with the combined effects of reduced plastic structure formation and a greater contribution from the soluble matrix.

The mechanical properties of the CCD formulations support this interpretation. Formulations containing higher MCC fractions generally showed higher hardness and tensile strength, whereas lactose-rich formulations showed lower mechanical strength. This is consistent with the positive coefficient of A (MCC/LM) in the quadratic regression equation (Table 15), confirming the dominant contribution of MCC to compact strength. This trend indicates that MCC contributed more strongly to compact integrity, while lactose-rich systems were mechanically weaker and more prone to structural loss during liquid exposure. The texture analyzer-derived RH response therefore provided a structural counterpart to the mechanical strength data: formulations that formed stronger and more persistent compacts tended to retain higher residual structure, whereas weaker and more soluble matrices tended to collapse more readily. However, the relationship between Heckel behavior and texture analyzer-derived disintegration descriptors should not be treated as a one-to-one correlation. Heckel analysis describes densification behavior during compression, whereas texture analyzer testing describes the response of the compacted matrix after exposure to liquid under controlled force. These are related but distinct processes. A material that densifies plastically may contribute to tablet strength and residual structure, but the final disintegration behavior also depends on porosity, liquid penetration, swelling pressure, soluble fraction, superdisintegrant distribution, and balance between matrix bonding and matrix weakening. Therefore, Heckel analysis should be considered supportive rather than definitive.

This caution is especially important for interpreting the superdisintegrants. Polyplasdone^®^ XL showed the lowest apparent yield pressure among the evaluated materials, indicating a strong densification tendency under compression. However, its texture analyzer profile also showed rapid swelling and efficient transition toward structural loss. This means that plastic densification behavior alone does not determine disintegration performance. For superdisintegrants, liquid uptake, swelling kinetics, strain recovery, and interaction with the surrounding tablet matrix are at least as important as compression behavior. Therefore, the Heckel results of Polyplasdone^®^ XL support its ability to participate in compact formation, but its favorable disintegration-related behavior is better explained by the combined TA-derived profile of high SE, negative RH, and high STE.

Similarly, Vivasol^®^ showed a higher apparent yield pressure than the crospovidone grades, but its texture analyzer profile showed strong collapse-related behavior, as reflected by highly negative RH. This again demonstrates that Heckel parameters cannot be used alone to rank superdisintegrant functionality. Croscarmellose sodium and crospovidone differ in swelling behavior, hydration pattern, particle morphology, and matrix-disrupting mechanism; these factors are not fully represented by apparent yield pressure. Therefore, Heckel analysis is useful for contextualizing compression and densification tendencies, but it cannot replace direct liquid-exposure profiling when the objective is to understand disintegration-related structural behavior.

Another important methodological limitation is that the Heckel analysis in this study was based on out-of-die thickness measurements and an experimentally defined reference state, rather than true-density-based in-die densification measurements. Therefore, the calculated yield pressures should be interpreted as apparent comparative parameters, not as absolute material constants. This approach was sufficient for comparing the relative densification behavior of the selected excipients under the same experimental conditions, but it does not allow broad generalization beyond the present material set and compression protocol.

Despite these limitations, the Heckel results added useful support to the overall interpretation of the formulation data. The apparent densification tendency of Vivapur^®^ 200 was consistent with the higher mechanical strength and residual structure observed in MCC-rich formulations. The higher apparent yield pressure and soluble character of Tablettose^®^ 80 were consistent with the lower residue height and greater collapse tendency of lactose-rich formulations. The superdisintegrant results further showed that favorable ODT behavior cannot be inferred from compression behavior alone and requires profile-based liquid-exposure characterization.

Therefore, Heckel analysis should be viewed as a supportive bridge between raw-material compaction behavior and formulation-level texture analyzer responses. It helped explain why MCC-rich systems retained more structure and why lactose-rich systems were more prone to structural loss, but it did not independently prove the disintegration mechanism. The integrated interpretation is that deformation behavior, matrix composition, and liquid-mediated structural transition act together to determine the texture analyzer profile of the final ODT formulation.

### 4.6. Comparative Kinetic Description of Texture Analyzer Profile Evolution

Comparative kinetic modeling was applied to the texture analyzer distance–time profiles as a secondary interpretive tool to describe the time-dependent evolution of the swelling and structural transition phases. The purpose of this analysis was not to assign a definitive disintegration mechanism or to directly transfer drug-release kinetic models to tablet disintegration behavior. Instead, the models were used to determine whether different regions of the distance–time profile showed distinct mathematical patterns. Therefore, the terms zero-order, first-order-like, Higuchi-type, Hixson–Crowell-type, and Korsmeyer–Peppas-type behavior should be understood as descriptive labels for profile similarity rather than as proof of a specific physical disintegration or drug-release mechanism [32,33,50].

Within the present texture analyzer framework, each model was assigned an adapted comparative meaning. Zero-order fitting was interpreted as an approximately constant-rate profile change within the selected segment. First-order-like fitting was interpreted as a profile change dependent on the remaining structural displacement or residual matrix response. Higuchi-type fitting was interpreted as square-root-of-time-like profile evolution, compatible with progressive liquid penetration, hydration, or swelling-front development, without proving these processes directly. Hixson–Crowell-type fitting was interpreted as geometry-related profile transformation, potentially reflecting changes in the dimensions, shape, or residual structure of the hydrated matrix during post-swelling transition. Korsmeyer–Peppas fitting was used only as an empirical power-law descriptor of profile evolution.

The swelling phase was most frequently described by Higuchi-type fitting. This pattern was observed for the center-point formulations F1, F3, F4, F8, and F10, all containing 50% MCC in the diluent phase and 6.25% Polyplasdone^®^ XL. The repeated occurrence of the same best-fit pattern across these center-point formulations supports the repeatability of the swelling-phase profile shape under the central formulation conditions. Higuchi-type fitting was also observed for F2, F11, F12, and F13, indicating that square-root-of-time-like swelling profile evolution was not limited to the center of the formulation space. In practical terms, this suggests that, for many formulations, the initial upward probe displacement evolved in a progressive penetration- or hydration-like manner; however, this should be interpreted only as mathematical similarity because direct liquid-front movement or internal hydration was not measured.

The edge and non-central formulations provided the most useful information for understanding formulation-dependent shifts in profile evolution. During the swelling phase, F5 and F9 shifted from the dominant Higuchi-type pattern toward Hixson–Crowell-type fitting. F5 combined a high MCC fraction with a high Polyplasdone^®^ XL concentration, whereas F9 combined a low MCC fraction with a low Polyplasdone^®^ XL concentration. This suggests that both a strong structure-forming matrix with high superdisintegrant content and a lactose-rich matrix with limited superdisintegrant content may alter the swelling trajectory toward a more geometry-related profile change. F6, containing high MCC and low Polyplasdone^®^ XL, showed first-order-like swelling behavior, suggesting a swelling trajectory more influenced by the remaining compact structure. F7, the most lactose-rich formulation, showed zero-order-like swelling behavior, indicating a more constant-rate upward displacement during the selected swelling segment. These deviations suggest that the swelling profile did not depend on a single factor alone, but on the combined MCC/lactose balance and Polyplasdone^®^ XL level.

The structural transition phase showed a different dominant pattern. Hixson–Crowell-type fitting most frequently provided the closest mathematical description, including for the center-point formulations and for F7, F11, and F12. This indicates that the transition from maximum swelling toward the residual plateau often shifted from a penetration-like swelling pattern to a geometry-related post-swelling transformation pattern. In the present context, this shift is consistent with progressive rearrangement, weakening, collapse, or loss of the hydrated matrix under the applied force. However, it should not be interpreted as evidence of a classical Hixson–Crowell dissolution or erosion mechanism, because the fitted response was probe displacement rather than mass loss or drug release.

The deviations observed during the structural transition phase further supported the formulation-dependent nature of the profile evolution. F2 and F6, both MCC-rich formulations with positive RH values, showed zero-order-like transition profiles, suggesting a more uniform approach toward the residual plateau and greater residual structural persistence. In contrast, F7 and F12, both lactose-rich formulations with negative RH values, followed Hixson–Crowell-type transition behavior, indicating a geometry-related collapse-associated transition toward lower residual structure. F11, which contained the highest Polyplasdone^®^ XL level at the center MCC/lactose ratio, also followed Hixson–Crowell-type transition behavior, suggesting that high superdisintegrant content promoted a transition profile mathematically similar to geometry-related structural change even in a balanced diluent matrix. F5 and F13 showed first-order-like transition behavior, suggesting that, under these conditions, the post-swelling transition was more dependent on the remaining hydrated matrix response. F9 showed Higuchi-type transition behavior, indicating a more gradual square-root-of-time-like post-swelling change rather than a strongly collapse-dominated transition.

Overall, the comparative kinetic evaluation showed that the dominant mathematical profile pattern shifted from Higuchi-type behavior during swelling to Hixson–Crowell-type behavior during structural transition. This shift supports the segmentation of the texture analyzer profiles into swelling and post-swelling transition regions and explains why a single endpoint or a single whole-profile fit would not adequately describe the disintegration-related behavior. Nevertheless, the kinetic assignments should be interpreted cautiously because model selection was based on R^2^ values from linearized equations, and in some formulations the differences between competing models were small. Therefore, kinetic modeling was considered a secondary profile-shape analysis that complemented SE, RH, and STE, rather than an independent mechanistic proof.

### 4.7. Practical Implications, Limitations, and Future Perspectives

The findings of this study have practical implications for the development of directly compressed ODTs. Pharmacopoeial disintegration testing and dissolution testing remain essential for confirming product performance. However, the present results show that, once formulations already meet the pharmacopoeial disintegration requirement and exhibit rapid drug release under comparative dissolution conditions, these endpoint-based tests may provide limited discrimination between formulation compositions. In such cases, texture analyzer-derived distance–time profiles can provide an additional development tool by describing how the tablet structure responds to limited fluid exposure before the final endpoint is reached.

The main practical value of the proposed approach is therefore not failure detection, but formulation discrimination within an apparently successful design space. All CCD formulations met the pharmacopoeial disintegration requirement and showed rapid dissolution behavior under the modified method used in this study, but they differed in swelling efficiency, residue height, and structural transition efficiency. This means that two formulations may both be acceptable according to conventional endpoints while still following different liquid-mediated structural pathways. For ODT development, this distinction may be relevant because residual structure, hydrated mass persistence, and collapse behavior may influence mouthfeel, residue perception, and patient acceptability [51,52].

From a formulation-development perspective, SE, RH, and STE provide complementary information. SE can be used to compare the rate of early swelling response, RH can be used to identify whether the hydrated tablet retains a persistent structure or transitions toward collapse, and STE can be used as a time-normalized descriptor of this transition when interpreted together with RH. Therefore, these descriptors may help formulators select compositions that balance sufficient mechanical strength before administration with rapid structural breakdown after contact with limited fluid. This may be particularly useful for low-dose ODT formulations, where the excipient matrix predominantly governs tablet performance.

Although the CCD models were useful for identifying formulation-dependent trends, their predictive interpretation should be considered limited for some responses. Among the evaluated responses, swelling efficiency showed the strongest model adequacy, with a significant model, non-significant lack of fit, high adjusted R^2^, and good predicted R^2^. In contrast, residue height and structural transition efficiency showed significant formulation effects but also significant lack of fit, indicating that these models should be interpreted mainly as descriptive trend models rather than as robust predictive equations. Similarly, pharmacopoeial disintegration time and Q5 drug release did not show statistically significant overall model responses and had low or negative predicted R^2^ values. Therefore, the CCD analysis was used primarily to support comparative interpretation within the investigated formulation space, not to establish a fully predictive design-space model.

The TA-extended SeDeM-ODT approach also has value as a structured preformulation and formulation-comparison concept. By transforming SE, RH, and STE into SeDeM-compatible radius values, profile-derived information can be integrated into the same visual and numerical format used for conventional SeDeM and SeDeM-ODT evaluation. This may make the approach easier to apply during excipient screening and formulation comparison. However, the TA-derived radius values should be interpreted as a comparative extension rather than as validated absolute acceptance criteria. The normalization limits used in this study were derived empirically from the investigated superdisintegrants and selected reference excipients. Therefore, the resulting rSE, rRH, rSTE, and TA-extended IGCB values are most appropriate for relative comparison within the studied material set.

Several limitations should be acknowledged. First, bisoprolol fumarate was used as a low-dose model drug. Because the drug load was low, formulation behavior was governed mainly by the excipient matrix. This was useful for evaluating excipient-driven disintegration behavior, but it limits direct extrapolation to high-dose ODT systems, poorly soluble drugs, or APIs with strong mechanical or surface-property effects. Future studies should apply the same TA-derived descriptor framework to APIs with different dose levels, solubility, particle properties, and deformation behavior to determine whether the descriptors remain discriminatory across broader formulation types.

Second, the proposed TA-derived descriptors were developed under one defined low-volume liquid controlled-force condition. Changes in liquid volume may affect liquid availability and wetting, whereas changes in the applied force may influence probe–sample interaction and the magnitude or timing of the recorded displacement. Therefore, the sensitivity of SE, RH, and STE to controlled variations in liquid volume and applied force should be evaluated in dedicated robustness studies during further method validation. The method used a fixed water volume, applied force, and test geometry. These conditions provided reproducibility and allowed relative comparison, but they do not fully reproduce the oral cavity, where saliva volume, salivary composition, tongue pressure, residence time, patient variability, and in-mouth movement are more complex. Therefore, the TA method should be interpreted as a controlled comparative test rather than a direct simulation of in vivo oral disintegration.

Third, the relationship between TA-derived descriptors and sensory performance was not directly evaluated. RH and STE may be relevant to residual structure and collapse behavior, but the present study did not include mouthfeel testing, trained sensory evaluation, or in vivo disintegration assessment. Therefore, statements about patient perception should remain cautious. Future work should investigate whether RH, STE, or the full distance–time profile correlates with sensory attributes such as grittiness, residue, pastiness, or perceived disintegration speed.

Fourth, the TA-extended SeDeM-ODT normalization strategy requires broader validation. The current normalization limits were based on three superdisintegrants and two reference excipients, which provided a useful functional range for the present study. However, a more general framework would require additional materials, including other crospovidone grades, croscarmellose sodium grades, sodium starch glycolate, co-processed superdisintegrants, alternative diluents, and different compression pressures. Without such broader validation, the TA-derived radius values should not be used as universal material acceptance criteria.

Fifth, Heckel analysis was used only as a supportive material-level tool. The analysis was performed on pure excipient compacts using thickness-based apparent relative density rather than true-density-based measurements. Therefore, the calculated apparent yield pressure values provided comparative information on densification behavior under the present conditions, but they should not be treated as absolute deformation constants. Moreover, Heckel analysis was performed on pure materials, whereas texture analyzer testing of the CCD formulations involved multicomponent tablets. For this reason, the Heckel results support the interpretation of MCC-rich structural persistence and lactose-rich collapse tendency, but they do not independently prove the disintegration mechanism.

Sixth, the kinetic modeling approach should be considered exploratory. The models were applied to selected profile regions using linearized equations and R^2^-based best-fit selection. This approach was useful for comparing profile evolution, but it does not establish a definitive disintegration or drug-release mechanism. Future work could strengthen this analysis by using nonlinear regression, additional model-selection criteria, replicate-level fitting, independent liquid penetration measurements, and microstructural imaging to verify whether the mathematical profile patterns correspond to physical changes in the tablet matrix.

Another limitation is that the CCD stage focused on one selected superdisintegrant, Polyplasdone^®^ XL. This choice was justified by its stronger preformulation and TA-derived profile compared with the other investigated superdisintegrants. However, testing only one superdisintegrant in the formulation design means that the CCD stage cannot fully compare how different superdisintegrant chemistries behave in the same MCC/lactose design space. Future studies should include parallel formulation designs using Polyplasdone^®^ XL-10, Vivasol^®^, and other superdisintegrants to determine whether the same SE/RH/STE relationships apply across different disintegration mechanisms.

Despite these limitations, the proposed approach provides a useful development framework. The study demonstrates that original SeDeM and conventional SeDeM-ODT can be used for structured raw-material screening, while texture analyzer-derived descriptors can add profile-based information on disintegration-related structural behavior. CCD modeling then allows these descriptors to be evaluated systematically as formulation responses. This layered strategy is consistent with a quality-by-design approach because it links material attributes, formulation variables, tablet properties, and performance-related structural behavior.

Overall, the TA-derived SeDeM-ODT extension should be viewed as an empirical and mechanistically informative screening tool for formulation development. Its strongest contribution is the ability to reveal differences in swelling, residual structure, and structural transition behavior when conventional disintegration and modified dissolution endpoints are already satisfied. With further validation, including inter-day and inter-operator precision assessments, across broader excipient sets, drug loads, compression conditions, and sensory or in vivo-relevant endpoints, the approach could become a useful complement to conventional SeDeM-ODT and pharmacopoeial testing in the development of directly compressed ODTs.

### 4.8. Integrated Interpretation of the Proposed Evaluation Framework

The overall findings of this study support a layered interpretation of ODT development. The original SeDeM evaluation provided a structured raw-material screening tool for assessing direct-compression-related properties, but it was not intended to predict disintegration mechanism. Conventional SeDeM-ODT then added an endpoint-based disgregability factor, which was useful as a standardized reference but showed limited ability to describe the dynamic structural behavior of polymeric superdisintegrants under the present conditions.

The texture analyzer-derived extension addressed this limitation by adding profile-based descriptors to the SeDeM-ODT framework. SE, RH, and STE provided information on different stages of liquid-mediated structural response: early swelling development, residual structural persistence or collapse, and time-normalized transition toward residual structure loss. Among these descriptors, RH provided the most direct indicator of structural persistence, SE described the efficiency of swelling development, and STE served as a complementary time-normalized descriptor when interpreted together with RH.

At the formulation level, the CCD results showed why this additional profile-based layer is useful. All formulations met the pharmacopoeial disintegration requirement and showed rapid bisoprolol fumarate release under the modified comparative dissolution method. However, these conventional endpoints had limited discriminatory value within the investigated design space. In contrast, the texture analyzer-derived responses revealed formulation-dependent differences associated with MCC/LM ratio and Polyplasdone^®^ XL concentration. This indicates that formulations can appear similarly acceptable by endpoint testing while differing in their underlying liquid-mediated structural pathways.

The supportive analyses further strengthened this interpretation without replacing the primary TA-derived evidence. Heckel analysis provided comparative material-level context for understanding why MCC-rich systems tended to retain more structure, whereas lactose-rich systems were more prone to structural loss. Comparative kinetic modeling showed that swelling and structural transition phases followed different profile-shape patterns, supporting the segmentation of the texture analyzer response into distinct regions. However, both Heckel analysis and kinetic modeling should be viewed as supportive tools rather than definitive proof of disintegration mechanism.

Taken together, the proposed framework positions texture analyzer-derived descriptors as a complementary development tool within SeDeM-ODT-based formulation assessment. The approach does not replace pharmacopoeial disintegration or dissolution testing, but it adds mechanistically informative, profile-level discrimination when conventional endpoints are already satisfied. This makes the TA-extended SeDeM-ODT concept particularly relevant for optimizing directly compressed ODTs in which excipient-driven structure formation, swelling, and collapse must be balanced within a narrow performance space.

## 5. Conclusions

This study establishes a proof-of-concept texture analyzer-derived extension of the SeDeM-ODT framework for the comparative evaluation of directly compressed orodispersible tablets. Conventional SeDeM and SeDeM-ODT provided a structured basis for raw-material screening and endpoint-based disgregability assessment, whereas the incorporation of swelling efficiency, residue height, and structural transition efficiency added profile-level information on swelling development, residual structural persistence, and transition toward structural loss.

All CCD formulations met the pharmacopoeial disintegration requirement and showed rapid bisoprolol fumarate release under the applied comparative dissolution conditions. However, pharmacopoeial disintegration time and early dissolution showed limited formulation-level discrimination within the investigated design space. In contrast, the TA-derived descriptors retained sensitivity to changes in the MCC/LM ratio and Polyplasdone^®^ XL concentration and revealed structural differences that were not fully captured by the conventional endpoints. These findings demonstrate that formulations with similarly acceptable pharmacopoeial performance may nevertheless follow distinct liquid-mediated structural pathways.

The proposed TA-extended SeDeM-ODT approach should therefore be considered a pilot, empirical, and comparative formulation-development framework rather than a replacement for compendial testing or a universally validated scoring system. The integrated experimental and statistical framework may serve as a preliminary methodological basis for supporting the evaluation and interpretation of laboratory-derived disintegration data, while its principal contribution lies in improving formulation discrimination within an already compliant ODT design space. The present findings provide a foundation for extending the scope of the proposed approach; however, further investigations across broader API and excipient sets, different drug loads, tablet geometries, manufacturing technologies, compaction conditions, and texture analyzer settings, together with independent reproducibility and robustness assessments, are needed to evaluate its wider applicability.

## Figures and Tables

**Table 1:** Parameters, calculation methods, normalization limits, and transformation equations used in the SeDeM and SeDeM-ODT evaluations [22,23,24,25,26].

Incidence Factor	Parameter	Symbol	Unit	Calculation/Definition	Determination Method	Normalization Limits for V *	Transformation Equation (V→r)
Dimension	Bulk Density	Da	g/mL	m/Vbulk	Bulk density measurement, USP <616>	0–1	r = 10V
	Tapped Density	Dc	g/mL	m/Vtapped	Tapped density measurement, USP <616>	0–1	r = 10V
Compressibility	Inter-particle Porosity	Ie	—	(Dc − Da)/(Dc × Da)	Calculated from Da and Dc	0–1.2	r = (10V)/1.2
	Carr Index	IC	%	[(Dc − Da)/Dc] × 100	Calculated from Da and Dc	0–50	r = V/5
	Cohesion Index	Icd	N	Crushing strength of compacts prepared under standardized conditions	Tablet crushing strength test	0–200	r = V/20
Flowability/Powder Flow	Hausner Ratio	IH	—	Dc/Da	Calculated from Da and Dc	1–3	r = (30 − 10V)/2
	Angle of Repose	α	°	tan^−1^(h/r)	Angle of repose, USP <1174>	0–50	r = 10 − (V/5)
	Powder Flow	t″	s	Time required for powder to flow through a funnel	Flow through an orifice, USP <1174>	0–20	r = 10 − (V/2)
Lubricity/Stability	Loss on Drying	%HR	%	Weight loss after drying	Loss on drying, USP <731>	0–10	r = 10 − V
	Hygroscopicity	%H	%	Weight gain after storage under controlled relative humidity	Gravimetric method	0–20	r = 10 − (V/2)
Lubricity/Dosage	Particles <50 μm	%Pf	%	Powder fraction passing through a 50 μm sieve	Sieve analysis	0–50	r = 10 − (V/5)
	Homogeneity Index	Iθ	—	Homogeneity index calculated from particle-size distribution	Particle-size distribution analysis	0–0.02	r = 500V
Disgregability	Effervescence Time	DE	s	Time required for complete dispersion of compact	Conventional SeDeM-ODT disgregability test	0–200	r = 10 − (V/20)
	Disintegration Time with Disk	DCD	s	Disintegration time measured in the presence of disk	Disintegration test, Ph. Eur. 2.9.1	0–200	r = 10 − (V/20)
	Disintegration Time without Disk	DSD	s	Disintegration time measured without disk	Disintegration test, Ph. Eur. 2.9.1	0–200	r = 10 − (V/20)

V represents the experimentally measured or calculated value of each parameter, and r represents the corresponding normalized SeDeM radius value. Radius values were expressed on a 0–10 scale. Calculated radius values below 0 or above 10 were truncated to 0 and 10, respectively. A radius value of r ≥ 5 was considered acceptable at the individual parameter level. The original SeDeM evaluation included the first 12 parameters, whereas the conventional SeDeM-ODT evaluation included the 12 original SeDeM parameters plus the 3 disgregability parameters, namely DE, DCD, and DSD.

**Table 2:** SeDeM and SeDeM-ODT indices used for material evaluation [14,22,27,28].

Index	Equation	Acceptance Criterion	Description
Parameter Index	IP = Number of parameters with r ≥ 5/Total number of parameters	≥0.50	Ratio of acceptable parameters to the total number of evaluated parameters
Parameter Profile Index	IPP = Mean of all radius values	≥5.00	Mean radius value of the complete SeDeM or SeDeM-ODT profile
Index of Good Compressibility	IGC = IPP × f	≥5.00	Original index used for the original twelve-parameter SeDeM profile
Index of Good Compressibility and Bucodispersibility	IGCB = IPP × f	≥5.00	Original index used for the fifteen-parameter SeDeM-ODT profile
Reliability Factor	f = reliability coefficient (according to the number of parameters)	—	f = 0.952 for twelve parameters and f = 0.971 for fifteen parameters

**Table 3:** Texture analyzer-derived parameters proposed for SeDeM-compatible disgregability evaluation.

Factor/ Incidence	Parameter	Descriptor Symbol	Radius Symbol	Descriptor Unit	Normalization Limits for V	Transformation Equation (V→r)
Disgregability	Swelling efficiency	SE	rSE	mm/s	0–0.044	r = V/0.0044
	Structural transition efficiency	STE	rSTE	mm/s	(−0.0025)–0.0142	r = (V + 0.0025)/0.00167
	Residue height	RH	rRH	mm	(−4.46)–1.57	r = (1.57 − V)/0.602

The normalization limits for SE, STE and RH were empirically derived from the experimental range of the materials tested in this study (three superdisintegrants and reference excipients). These limits are dataset-specific and should not be interpreted as universal acceptance criteria. Broader validation with a wider range of excipients and compression conditions is required before these limits can be proposed as general SeDeM-compatible thresholds.

**Table 4:** Mathematical models used for comparative kinetic evaluation of texture analyzer distance–time profiles [32,33,34,35,36].

Model	Linearized Equation	Kinetic Parameter	Comparative Interpretation in This Study
Zero-order	$Q_{t}=Q_{0}+k_{0}t$	k_0_, R^2^	Constant-rate profile change
First-order	$\ln(Q_{\infty }-Q_{t})=lnQ_{\infty }-k_{1}t$	k_1_, R^2^	Profile change is dependent on the remaining structural response
Higuchi	$Q_{t}=k_{H}t^{\frac{1}{2}}$	$k_{H}$, R^2^	Diffusion/penetration-like profile progression
Hixson– Crowell	$Q_{\infty }^{\frac{1}{3}}-Q_{t}^{\frac{1}{3}}=k_{HC}t$	$k_{HC}$, R^2^	Geometry-related structural transformation
Korsmeyer– Peppas	$log\left(\frac{Q_{t}}{Q_{\infty }}\right)=logk_{KP}+n\;log(t)$	$k_{KP}$, n, R^2^	Empirical power-law description of profile evolution

In this table, $Q_{t}$ represents the normalized distance-based texture analyzer response at time t, and $Q_{0}$ or $Q_{\infty }$ represents the initial or reference value of the selected profile segment. The models were used only for comparative description of the evolution of distance–time profiles and were not intended to define a definitive drug-release or disintegration mechanism.

**Table 5:** Independent variables and levels used in the central composite design.

Factor	Independent Variable	Symbol	Axial Low	Low Level	Center Level	High Level	Axial High
X1	Vivapur^®^ 200 fraction in the diluent phase	MCC/LM	14.645	25	50	75	85.355
X2	Polyplasdone^®^ XL concentration	Superdisintegrant (%)	0.947	2.5	6.25	10	11.553

MCC/LM represents the percentage of Vivapur^®^ 200 within the total diluent fraction, with the remaining diluent fraction completed with Tablettose^®^ 80. Superdisintegrant (%) represents Polyplasdone^®^ XL concentration as a percentage of total tablet weight. Bisoprolol fumarate and magnesium stearate were kept constant at 5 mg and 3 mg per tablet, respectively.

**Table 6:** Quantitative composition of bisoprolol fumarate ODT formulations prepared according to the central composite design.

Formulation	MCC Fraction in Diluent Phase (%)	Polyplasdone^®^ XL (%)	Bisoprolol Fumarate (mg)	MgSt (mg)	Polyplasdone^®^ XL (mg)	Vivapur^®^ 200 (mg)	Tablettose^®^ 80 (mg)
F1	50.00	6.25	5.00	3.00	18.75	136.63	136.63
F2	85.36	6.25	5.00	3.00	18.75	233.23	40.02
F3	50.00	6.25	5.00	3.00	18.75	136.63	136.63
F4	50.00	6.25	5.00	3.00	18.75	136.63	136.63
F5	75.00	10.00	5.00	3.00	30.00	196.50	65.50
F6	75.00	2.50	5.00	3.00	7.50	213.37	71.13
F7	14.65	6.25	5.00	3.00	18.75	40.02	233.23
F8	50.00	6.25	5.00	3.00	18.75	136.63	136.63
F9	25.00	2.50	5.00	3.00	7.50	71.13	213.37
F10	50.00	6.25	5.00	3.00	18.75	136.63	136.63
F11	50.00	11.55	5.00	3.00	34.66	128.67	128.67
F12	25.00	10.00	5.00	3.00	30.00	65.50	196.50
F13	50.00	0.95	5.00	3.00	2.84	144.58	144.58

Values are rounded to two decimal places. MCC fraction represents the percentage of Vivapur^®^ 200 within the total diluent phase, with the remaining diluent fraction completed with Tablettose^®^ 80. Polyplasdone^®^ XL (%) represents the superdisintegrant concentration as a percentage of the total tablet weight. Magnesium stearate was fixed at 1% *w/w*, corresponding to 3 mg per 300 mg tablet. Minor differences in calculated totals may occur due to rounding.

**Table 7:** SeDeM radius values, factor scores, and indices of bisoprolol fumarate and selected excipients.

Excipient		Parameters (Radius)												Factor						INDEX	
		Da	Dc	Ie	IC	Icd	IH	(α)	T″	%HR	%H	%Pf	(Iθ)	Dimension	Compressibility	Flowability	Lubricity/Stability	Lubricity/Dosage	Parameter Index (IP)	Parameter profile Index (IPP)	Good Compressibility Index (IGC)
Super Disintegrants	Polyplasdone^®^ XL (Crospovidone type A)	2.97	3.95	6.94	4.95	4.77	8.35	4.28	8.66	5.83	0.00	10.00	1.53	3.46	5.55	7.10	2.92	5.77	0.42	5.19	4.94
	Polyplasdone^®^ XL-10 (Crospovidone type B)	3.03	4.48	8.89	6.46	5.90	7.61	0.00	0.00	3.22	1.07	9.03	0.62	3.76	7.08	2.54	2.15	4.83	0.42	4.19	3.99
	Vivasol (Croscarmellose Sodium)	5.22	6.52	3.19	3.99	0.03	8.75	0.00	0.00	5.40	4.86	0.00	0.10	5.87	2.40	2.92	5.13	0.05	0.33	3.17	3.02
Diluents	Vivapur^®^ 200 (MCC 200)	3.37	4.58	6.53	5.28	8.73	8.21	0.00	0.00	7.64	7.95	6.16	0.40	3.98	6.85	2.74	7.80	3.28	0.58	4.90	4.67
	Avicel PH 102 (MCC 102)	3.24	4.51	7.22	5.62	7.84	8.04	0.00	0.00	6.25	8.25	5.49	1.50	3.88	6.89	2.68	7.25	3.50	0.58	4.83	4.60
	Tablettose^®^ 80 (α-lactose monohydrate)	5.63	7.32	3.40	4.60	0.27	8.51	3.34	9.30	9.82	10.00	8.15	1.08	6.48	2.76	7.05	9.91	4.62	0.58	5.95	5.67
API	Bisoprolol fumarate	2.96	5.00	10.00	8.18	3.49	6.54	0.00	0.00	9.26	9.87	9.95	2.28	3.98	7.22	2.18	9.57	6.12	0.58	5.63	5.36

**Table 8:** Conventional SeDeM-ODT evaluation of the investigated superdisintegrants.

Excipient		Parameters (Radius)																Factor						INDEX	
		Da	Dc	Ie	IC	Icd	IH	(α)	T″	%HR	%H	%Pf	(Iθ)	DE	DCD	DSD	Dimension	Compressibility	Flowability	Lubricity/Stability	Lubricity/Dosage	Disgregability	Parameter Index (IP)	Parameter profile Index (IPP)	IGCB (Index of Good Compressibility and Bucodispersibility)
Super Disintegrants	Polyplasdone^®^ XL (Crospovidone type A)	2.97	3.95	6.94	4.95	4.77	8.35	4.28	8.66	5.83	0.00	10.00	1.53	0.00	5.02	0.00	3.46	5.55	7.10	2.92	5.77	1.67	0.40	4.48	4.35
	Polyplasdone^®^ XL-10 (Crospovidone type B)	3.03	4.48	8.89	6.46	5.90	7.61	0.00	0.00	3.22	1.07	9.03	0.62	0.00	0.00	0.00	3.76	7.08	2.54	2.15	4.83	0.00	0.33	3.35	3.25
	Vivasol (Croscarmellose Sodium)	5.22	6.52	3.19	3.99	0.03	8.75	0.00	0.00	5.40	4.86	0.00	0.10	0.00	4.06	0.00	5.87	2.40	2.92	5.13	0.05	1.35	0.27	2.80	2.72

**Table 9:** Texture analyzer-derived descriptors of pure superdisintegrants and reference excipients.

Material	Dmax (mm)	tA (s)	RH (mm)	tB (s)	SE	STE
Polyplasdone^®^ XL	1.5020 ± 0.0300	34.4670 ± 2.5720	−4.0250 ± 0.0890	282.8000 ± 1.7090	0.0436	0.0142
Polyplasdone^®^ XL-10	1.0930 ± 0.0320	218.9330 ± 5.8590	−2.5580 ± 0.8210	1079.8670 ± 268.3200	0.0050	0.0024
Vivasol^®^	1.5960 ± 0.0950	47.3330 ± 3.5350	−4.4610 ± 0.1860	621.1330 ± 71.3280	0.0337	0.0072
Tablettose^®^ 80	0.0000 ± 0.0000	0.0000 ± 0.0000	−1.4480 ± 0.2660	210.1330 ± 100.7340	0.0000	0.0069
Vivapur^®^ 200	1.5410 ± 0.0170	599.0000 ± 0.7210	1.5690 ± 0.0380	628.8000 ± 51.4470	0.0026	−0.0025

**Table 10:** Texture analyzer-derived SeDeM-ODT evaluation of the investigated superdisintegrants.

Excipient		Parameters (Radius)															Factor						INDEX		
		Da	Dc	Ie	IC	Icd	IH	(α)	T″	%HR	%H	%Pf	(Iθ)	rSE	rSTE	rRH	Dimension	Compressibility	Flowability	Lubricity/Stability	Lubricity/Dosage	Disgregability	Parameter Index (IP)	Parameter Profile Index (IPP)	IGCB (Index of Good Compressibility and Bucodispersibility)
Super Disintegrants	Polyplasdone^®^ XL (Crospovidone type A)	2.97	3.95	6.94	4.95	4.77	8.35	4.28	8.66	5.83	0.00	10.00	1.53	9.90	10.00	9.29	3.46	5.55	7.10	2.92	5.77	9.73	0.60	6.09	5.91
	Polyplasdone^®^ XL-10 (Crospovidone type B)	3.03	4.48	8.89	6.46	5.90	7.61	0.00	0.00	3.22	1.07	9.03	0.62	1.13	2.91	6.86	3.76	7.08	2.54	2.15	4.83	3.63	0.40	4.08	3.96
	Vivasol (Croscarmellose Sodium)	5.22	6.52	3.19	3.99	0.03	8.75	0.00	0.00	5.40	4.86	0.00	0.10	7.66	5.80	10.00	5.87	2.40	2.92	5.13	0.05	7.82	0.40	4.10	3.98

**Table 11:** Physical, mechanical, and disintegration properties of bisoprolol fumarate ODT formulations.

Sample	Weight (mg) (n = 20)	Diameter (mm) (n = 10)	Thickness (mm) (n = 10)	Hardness (N) (n = 10)	Tensile Strength (MPa)	Friability (%)	Disintegration Time (s) (n = 3)
F1	300.28 ± 1.139	12.15 ± 0.005	2.16 ± 0.010	80.48 ± 3.147	1.95 ± 0.08	0.23	15.31
F2	304.09 ± 1.380	12.15 ± 0.006	2.07 ± 0.010	159.99 ± 3.515	4.05 ± 0.09	0.02	24.79
F3	299.96 ± 1.347	12.15 ± 0.005	2.16 ± 0.008	79.36 ± 2.648	1.93 ± 0.06	0.25	15.96
F4	299.56 ± 1.358	12.15 ± 0.005	2.15 ± 0.010	79.44 ± 3.218	1.94 ± 0.08	0.21	15.64
F5	299.18 ± 1.544	12.15 ± 0.006	2.16 ± 0.009	138.79 ± 2.858	3.37 ± 0.07	0.03	35.47
F6	301.02 ± 1.444	12.15 ± 0.007	2.04 ± 0.010	197.87 ± 3.106	5.08 ± 0.08	0.02	99.27
F7	301.75 ± 2.829	12.15 ± 0.006	2.17 ± 0.008	56.23 ± 3.290	1.36 ± 0.08	0.59	21.91
F8	300.60 ± 1.487	12.15 ± 0.005	2.16 ± 0.010	79.88 ± 2.069	1.94 ± 0.05	0.21	16.02
F9	301.95 ± 2.829	12.15 ± 0.008	2.13 ± 0.007	62.77 ± 4.690	1.54 ± 0.12	0.6	19.59
F10	300.31 ± 1.491	12.15 ± 0.005	2.15 ± 0.008	80.40 ± 2.841	1.96 ± 0.07	0.27	14.92
F11	301.19 ± 1.657	12.15 ± 0.006	2.16 ± 0.006	87.47 ± 3.215	2.12 ± 0.08	0.24	16.91
F12	300.59 ± 2.684	12.15 ± 0.004	2.18 ± 0.010	58.82 ± 5.039	1.41 ± 0.12	0.42	12.24
F13	302.78 ± 1.652	12.15 ± 0.006	2.12 ± 0.027	84.18 ± 3.892	2.08 ± 0.10	0.29	40.86

**Table 12:** Cumulative dissolution performance of bisoprolol fumarate ODT formulations.

Sample	Q5 (%)	Q10 (%)	Q15 (%)	Q30 (%)
F1	72.28 ± 10.69	93.24 ± 0.75	104.20 ± 6.97	102.83 ± 7.76
F2	75.70 ± 7.92	91.51 ± 8.47	106.85 ± 7.48	103.76 ± 6.77
F3	75.06 ± 9.22	94.02 ± 0.73	101.57 ± 7.92	103.04 ± 6.69
F4	73.36 ± 9.33	95.19 ± 0.65	100.80 ± 6.98	102.35 ± 5.78
F5	80.52 ± 2.46	94.92 ± 8.64	101.25 ± 4.49	104.73 ± 6.59
F6	62.51 ± 1.32	84.09 ± 2.24	108.17 ± 3.43	111.07 ± 0.71
F7	73.18 ± 3.57	91.90 ± 12.27	102.67 ± 5.90	106.14 ± 1.88
F8	73.15 ± 8.40	91.50 ± 0.37	101.80 ± 1.58	103.62 ± 1.48
F9	67.55 ± 0.84	96.26 ± 0.92	104.99 ± 1.20	106.32 ± 1.21
F10	74.08 ± 8.91	96.07 ± 3.41	99.10 ± 1.81	101.87 ± 0.98
F11	76.62 ± 2.53	101.74 ± 15.73	105.06 ± 10.92	103.64 ± 6.44
F12	73.84 ± 10.45	94.26 ± 3.56	104.54 ± 11.21	105.98 ± 11.30
F13	76.34 ± 5.38	95.13 ± 8.05	110.72 ± 0.17	108.19 ± 2.26

**Table 13:** Texture analyzer-derived disintegration descriptors of bisoprolol fumarate ODT formulations. (n = 3).

Sample	Dmax (mm)	tA (s)	tB (s)	RH (mm)	SE (mm/s)	STE (mm/s)
F1	0.948 ± 0.068	16.133 ± 3.700	272.133 ± 46.149	0.462 ± 0.007	0.05876	−0.00170
F2	2.214 ± 0.009	167.800 ± 20.987	262.200 ± 35.226	1.965 ± 0.031	0.01320	−0.00749
F3	0.945 ± 0.059	15.067 ± 2.928	469.333 ± 149.662	0.268 ± 0.039	0.06270	−0.00057
F4	0.952 ± 0.027	15.600 ± 0.721	260.667 ± 32.411	0.475 ± 0.016	0.06103	−0.00182
F5	1.667 ± 0.085	49.800 ± 4.812	420.067 ± 117.322	−0.121 ± 0.061	0.03347	0.00029
F6	1.421 ± 0.001	94.267 ± 19.344	147.600 ± 43.574	1.359 ± 0.037	0.01508	−0.00921
F7	0.082 ± 0.014	7.467 ± 0.611	257.667 ± 14.300	−0.311 ± 0.018	0.01103	0.00121
F8	0.873 ± 0.058	18.333 ± 1.514	294.867 ± 18.218	0.356 ± 0.023	0.04764	−0.00121
F9	0.233 ± 0.013	8.867 ± 0.577	550.267 ± 17.436	−0.024 ± 0.003	0.02624	0.00004
F10	0.875 ± 0.048	16.333 ± 1.172	436.733 ± 163.701	0.292 ± 0.049	0.05357	−0.00067
F11	0.799 ± 0.064	14.000 ± 1.200	356.133 ± 137.573	−0.209 ± 0.029	0.05705	0.00059
F12	0.257 ± 0.015	8.333 ± 0.702	197.467 ± 13.985	−0.394 ± 0.003	0.03084	0.00200
F13	0.916 ± 0.017	30.133 ± 4.868	66.933 ± 12.980	0.852 ± 0.019	0.03040	−0.01272

Dmax and tA represent the maximum swelling distance and the time required to reach maximum swelling, respectively. RH and tB represent the residue height and the time corresponding to the residual plateau region. SE was calculated as Dmax/tA, and STE was calculated as −RH/tB. Positive RH values therefore produce negative STE values, indicating residual structural persistence, whereas negative RH values produce positive STE values, indicating transition toward structural collapse or loss of residual structure. SE, RH, and STE values were used as CCD response variables. Values are expressed as mean ± standard deviation (n = 3).

**Table 14:** Summary of statistical model evaluation for CCD responses.

Response	Selected Model	Model *p*-Value	Significant Terms	Lack of Fit *p*-Value	Adjusted R^2^	Predicted R^2^	Adeq. Precision
Tensile strength	Quadratic	0.0011	A, A^2^	<0.0001	0.8603	0.4209	11.6428
Swelling efficiency	Quadratic	0.0001	B, A^2^, B^2^	0.7444	0.9224	0.8683	12.7864
Residue height	Linear	0.0005	A, B	0.0053	0.7428	0.5338	12.3925
Structural transition efficiency	Linear	0.0009	A, B	0.0028	0.7043	0.4930	11.4138
Disintegration time	Linear	0.0606	None	<0.0001	0.3150	-0.1596	5.7077
Q5 drug release (%)	2FI	0.1006	B	0.0057	0.3109	-0.9665	5.9083

**Table 15:** Selected response surface models and coded equations for the central composite design responses.

Response	Selected Model	Coded Equation
Tensile Strength (MPa)	Quadratic	1.97 + 1.18A − 0.2267B − 0.4025AB + 0.4982A^2^ + 0.1958B^2^
Swelling Efficiency (mm/s)	Quadratic	0.0567 − 0.0007A + 0.0076B + 0.0034AB − 0.0227A^2^ − 0.0069B^2^
Residue Height (mm)	Linear	0.3823 + 0.6094A − 0.4187B
Structural Transition Efficiency (mm/s)	Linear	−0.0024 − 0.0029A + 0.0038B

A represents the coded MCC/LM ratio, and B represents the coded superdisintegrant concentration (%). Equations are expressed in coded factor units.

**Table 16:** Heckel parameters of the investigated excipients.

Material	K	A	Py (MPa)	R^2^
Avicel PH 102/MCC 102	0.115	0.5137	8.66	0.9735
Vivapur^®^ 200/MCC 200	0.1390	0.4950	7.19	0.9905
Tablettose^®^ 80/α-lactose monohydrate	0.0975	0.8794	10.26	0.9650
Vivasol/croscarmellose sodium	0.1130	0.2383	8.85	0.9849
Polyplasdone^®^ XL	0.1611	0.2014	6.21	0.9894
Polyplasdone^®^ XL-10	0.1489	0.8885	6.72	0.9596

**Table 17:** Comparative kinetic model fitting of the swelling phase of texture analyzer profiles.

R^2^—Swelling Phase							
Sample	Zero-Order	First-Order	Higuchi	Hixson–Crowell	Korsmeyer–Peppas	Best-Fit Model	k
F1	0.989	0.965	0.999	0.985	0.785	Higuchi	0.42960
F2	0.946	0.969	0.993	0.984	0.730	Higuchi	0.09138
F3	0.985	0.964	0.999	0.986	0.789	Higuchi	0.43900
F4	0.988	0.959	0.999	0.986	0.797	Higuchi	0.44303
F5	0.938	0.972	0.972	0.982	0.761	Hixson–Crowell	0.01747
F6	0.883	0.970	0.948	0.970	0.779	First-order	0.07000
F7	0.919	0.864	0.879	0.915	0.406	Zero-order	0.21983
F8	0.979	0.976	0.996	0.993	0.786	Higuchi	0.37937
F9	0.944	0.959	0.957	0.982	0.575	Hixson–Crowell	0.14259
F10	0.973	0.916	0.998	0.961	0.791	Higuchi	0.39630
F11	0.969	0.937	0.997	0.970	0.780	Higuchi	0.44744
F12	0.956	0.954	0.985	0.984	0.631	Higuchi	0.80715
F13	0.978	0.974	0.988	0.987	0.789	Higuchi	0.28153

**Table 18:** Comparative kinetic model fitting of the structural transition phase of texture analyzer profiles.

R^2^—Structural Transition Phase							
Sample	Zero-Order	First-Order	Higuchi	Hixson–Crowell	Korsmeyer–Peppas	Best-Fit Model	k
F1	0.977	0.988	0.994	0.997	0.790	Hixson–Crowell	0.00157
F2	0.986	0.924	0.966	0.955	0.746	Zero-order	0.01396
F3	0.981	0.990	0.996	0.998	0.832	Hixson–Crowell	0.00084
F4	0.981	0.980	0.993	0.997	0.792	Hixson–Crowell	0.00160
F5	0.855	0.951	0.922	0.930	0.861	First-order	0.01489
F6	0.989	0.907	0.962	0.950	0.804	Zero-order	0.01551
F7	0.983	0.974	0.996	0.996	0.750	Hixson–Crowell	0.00156
F8	0.982	0.980	0.994	0.998	0.770	Hixson–Crowell	0.00133
F9	0.992	0.976	0.998	0.995	0.701	Higuchi	0.02269
F10	0.967	0.990	0.988	0.997	0.767	Hixson–Crowell	0.00098
F11	0.916	0.972	0.969	0.996	0.805	Hixson–Crowell	0.00095
F12	0.858	0.975	0.939	0.996	0.763	Hixson–Crowell	0.00174
F13	0.984	0.988	0.983	0.984	0.810	First-order	0.09885

**Figure A1:**
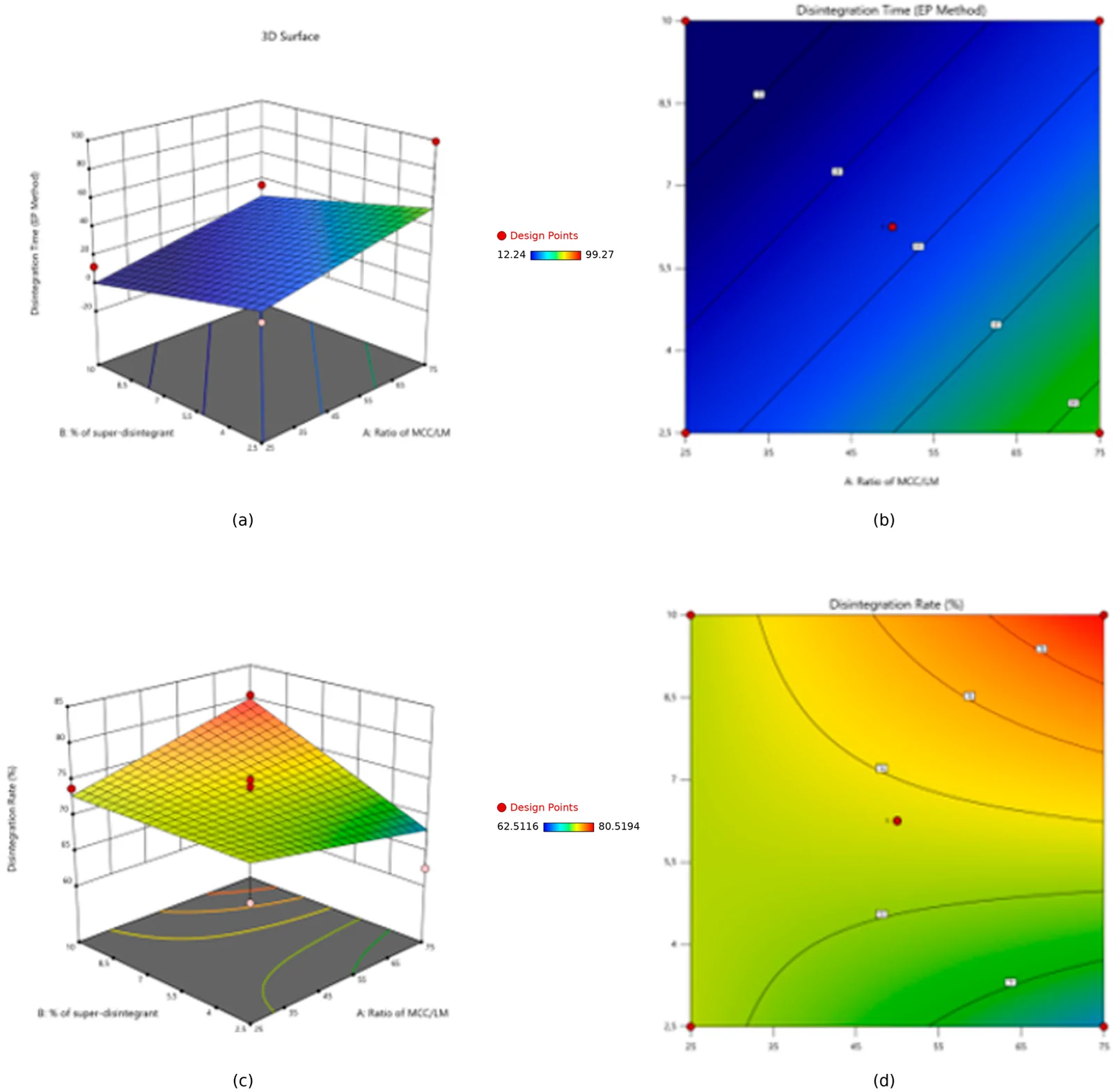
Exploratory response surface and contour plots for conventional endpoint responses with limited predictive model performance: (**a**) response surface plot of pharmacopoeial disintegration time; (**b**) contour plot of pharmacopoeial disintegration time; (**c**) response surface plot of Q5 drug release; and (**d**) contour plot of Q5 drug release. These plots are provided for transparency only. Because the corresponding models were not statistically significant and showed poor predictive performance and/or significant lack of fit, they were not used for formulation optimization or mechanistic interpretation.

## Data Availability

Data presented in this study are contained within the article. Further inquiries can be directed to the corresponding author.

## Abbreviations

The following abbreviations are used in this manuscript:

2FI	Two-factor interaction
ANOVA	Analysis of variance
API	Active pharmaceutical ingredient
CCD	Central composite design
CCS	Croscarmellose sodium
DCD	Disintegration time with disk
DE	Effervescence/dispersion time
Dmax	Maximum swelling distance
DSD	Disintegration time without disk
IGC	Index of good compressibility
IGCB	Index of good compressibility and bucodispersibility
IP	Parameter index
IPP	Parameter profile index
LM	Lactose monohydrate
MCC	Microcrystalline cellulose
MCC/LM	Microcrystalline cellulose/lactose monohydrate ratio
MgSt	Magnesium stearate
ODT	Orodispersible tablet
Ph. Eur.	European Pharmacopoeia
Py	Apparent yield pressure
Q5	Cumulative drug release at 5 min
Q10	Cumulative drug release at 10 min
Q15	Cumulative drug release at 15 min
Q30	Cumulative drug release at 30 min
R^2^	Coefficient of determination
RH	Residue height
rRH	Normalized SeDeM-compatible radius value of residue height
rSE	Normalized SeDeM-compatible radius value of swelling efficiency
rSTE	Normalized SeDeM-compatible radius value of structural transition efficiency
SE	Swelling efficiency
SeDeM	SeDeM expert system
SeDeM-ODT	SeDeM-ODT expert system
STE	Structural transition efficiency
TA	Texture analyzer
tA	Time to maximum swelling
tB	Time to residual plateau
USP	United States Pharmacopeia
UV	Ultraviolet
w/w	Weight by weight
